# Pharmacologically increasing cGMP improves proteostasis and reduces neuropathy in mouse models of CMT1

**DOI:** 10.1007/s00018-024-05463-1

**Published:** 2024-10-14

**Authors:** Seth M. Moore, Joseph Gawron, Mckayla Stevens, Leandro N. Marziali, Emmanuel S. Buys, G. Todd Milne, Maria Laura Feltri, Jordan J.S. VerPlank

**Affiliations:** 1 Department of Biochemistry, Institute for Myelin and Glia Exploration, Jacobs School of Medicine and Biomedical Sciences, State University of New York at Buffalo, Buffalo, NY 14203 USA; 2 IRCCS Neurological institute ‘Carlo Besta’, Milano, Italy; 3 Department of Medical Biotechnology and Translational Medicine, Universita’ degli Studi di Milano, Milano, Italy; 4 Cyclerion Therapeutics, 245 First Street Riverview II, 18th floor, Cambridge, MA 02142 USA; 5 Department of Anatomy, Physiology, and Genetics, F. Edward Hebert School of Medicine, Uniformed Services University of the Health Sciences, Bethesda, MD 20814 USA

**Keywords:** Proteasome, Protein degradation, cGMP, Peripheral neuropathy, Phosphorylation, Charcot Marie Tooth

## Abstract

Increasing cyclic GMP activates 26S proteasomes via phosphorylation by Protein Kinase G and stimulates the intracellular degradation of misfolded proteins. Therefore, agents that raise cGMP may be useful therapeutics against neurodegenerative diseases and other diseases in which protein degradation is reduced and misfolded proteins accumulate, including Charcot Marie Tooth 1A and 1B peripheral neuropathies, for which there are no treatments. Here we increased cGMP in the S63del mouse model of CMT1B by treating for three weeks with either the phosphodiesterase 5 inhibitor tadalafil, or the brain-penetrant soluble guanylyl cyclase stimulator CYR119. Both molecules activated proteasomes in the affected peripheral nerves, reduced polyubiquitinated proteins, and improved myelin thickness and nerve conduction. CYR119 increased cGMP more than tadalafil in the peripheral nerves of S63del mice and elicited greater biochemical and functional improvements. To determine whether raising cGMP could be beneficial in other neuropathies, we first showed that polyubiquitinated proteins and the disease-causing protein accumulate in the sciatic nerves of the C3 mouse model of CMT1A. Treatment of these mice with CYR119 reduced the levels of polyubiquitinated proteins and the disease-causing protein, presumably by increasing their degradation, and improved myelination, nerve conduction, and motor coordination. Thus, pharmacological agents that increase cGMP are promising treatments for CMT1 neuropathies and may be useful against other proteotoxic and neurodegenerative diseases.

**Supplementary Information:**

The online version contains supplementary material available at 10.1007/s00018-024-05463-1.

## Introduction

Charcot Marie Tooth (CMT) neuropathies are collectively the most common inherited neurological diseases in the world with an estimated global prevalence of one person per 2,500 in the population. There are no curative treatments. The most prevalent subtype of CMT diseases is CMT1, which are demyelinating neuropathies. Of these, the most common are CMT1A and CMT1B, which constitute ~ 40% and 6% of all CMT cases, respectively [1]. CMT1A is caused by the duplication of peripheral myelin protein 22 (PMP22), resulting in its overexpression, and CMT1B is caused by mutations in myelin protein zero (MPZ) [2].

MPZ is synthesized by Schwann cells, the myelinating glia of the peripheral nervous system, and comprises approximately 50% of the total protein content of peripheral nerves [3]. Over 200 mutations in MPZ have been reported to cause CMT neuropathies [4]. The deletion of serine 63 in MPZ (MPZ^S63del^) causes CMT1B through gain of toxic function mechanisms. In S63del transgenic mice, which express MPZ^S63del^ under the control of the endogenous promoter, the mutant protein accumulates in the endoplasmic reticulum (ER) of Schwann cells, where it induces a canonical Unfolded Protein Response (UPR) [5, 6]. Furthermore, protein degradation by the 26S proteasome is reduced in the peripheral nerves of these mice [7].

In the Ubiquitin Proteasome System (UPS), proteins to be degraded are first conjugated to ubiquitin, and then bound, unfolded, and hydrolyzed into small peptides by 26S proteasomes [8]. This pathway degrades the vast majority of proteins in mammalian cells, including those that are misfolded and mutated (e.g., MPZ^S63del^). When there is a decrease in protein breakdown by proteasomes, polyubiquitinated proteins accumulate in the affected cells and disrupt protein homeostasis [9]. In addition to S63del mice, such impairment in proteasomal function has been reported in vertebrate models of several diseases, especially neurodegenerative diseases, that are caused by the expression of mutant, aggregation-prone proteins [10].

The breakdown of polyubiquitinated proteins by 26S proteasomes is tightly regulated [11]. One newly appreciated mechanism of increasing the proteasomal degradation of some proteins is phosphorylation [12]. Three kinases have been shown to increase proteasomal protein degradation in cells via phosphorylation of distinct proteasome components: DYRK2 [13], cAMP-dependent kinase (PKA) [14, 15], and cGMP-dependent kinase (PKG) [16]. Activating these kinases and stimulating protein degradation by proteasomes could be a new therapeutic approach to treat diseases in which there is a decrease in protein breakdown by the UPS. In fact, pharmacological agents that raise cAMP or cGMP have been shown to activate proteasomes, increase the degradation of the disease-causing protein, decrease protein accumulation, and reduce the associated pathology in vertebrate models of diseases in which there is evidence of proteasome impairment – tauopathies [16, 17], hereditary cardiomyopathies [18, 19], and CMT1B hereditary peripheral neuropathies [20].

cGMP is an important signaling molecule that is synthesized by soluble guanylyl cyclase (sGC) in response to nitric oxide (NO). It is broken down to GMP by phosphodiesterases (PDEs). cGMP influences many cellular processes via activation of PKG, the most studied of which is the relaxation of smooth muscles [21]. Two main classes of pharmacological agents that increase intracellular concentrations of cGMP via the NO pathway have been approved by the Food and Drug Administration (FDA) and are widely used in medicine: PDE5 inhibitors (e.g., sildenafil and tadalafil), which are used to treat erectile dysfunction and pulmonary arterial hypertension [22], and sGC stimulators (e.g., riociguat and vericiguat), which are used to treat pulmonary arterial hypertension and cardiac failure [23]. Compounds in both classes have favorable safety and tolerability profiles when administered acutely and chronically.

We recently reported that increasing cGMP in S63del mice by treating with sildenafil intraperitoneally for two weeks activated proteasomal activity in the sciatic nerves, reduced the levels of polyubiquitinated proteins and markers of the UPR, and restored myelin thickness and nerve conduction [20]. Here we investigated whether compounds with more favorable pharmacodynamics than sildenafil, delivered *per os*, could improve proteostasis, myelination, and nerve conduction. We treated the S63del mouse model of CMT1B with tadalafil, a PDE5 inhibitor with a longer half-life in vivo than sildenafil, or CYR119 [24], a novel brain-penetrant sGC stimulator, and evaluated proteostasis in the sciatic nerves, levels of cGMP, and morphological and functional indicators of neuropathy. Encouraged by the results we obtained in the S63del mouse model of CMT1B, we then investigated whether compounds that increase cGMP could also have therapeutic effects in the C3 mouse model of CMT1A neuropathy.

## Methods

### Animal models

Management of the mouse colony and all experiments involving mice were conducted in strict adherence to the protocols approved by the Institutional Animal Care and Use Committees (IACUC) of the Roswell Park Cancer Institute and the State University of New York at Buffalo. All mice were housed in pathogen-free conditions with *ad libitum* access to food and water in filter-top cages maintained at 70 °F, 50% humidity, and exposed to a 12-hour light/12-hour dark cycle. S63del mice [25] were maintained in a congenic FVB background. C3 mice, PMP22-overexpressing B6.Cg-Tg(PMP22)C3Fbas/J, were obtained from Jackson Laboratories (Strain #:030052, RRID: IMSR_JAX:030052) and maintained in a congenic C57BL/6 background. PMP22 knockout mice (RRID: MGI:3794448) [26] were maintained in a congenic C57BL/6 background. The transgenes were maintained in hemizygosity in both C3 and S63del mice. Male and female mice were used for all experiments and littermates were compared whenever possible.

To determine the genotypes of the transgenic mice, tail genomic DNA was extracted using NaOH/EDTA or phenol chloroform, and PCR was performed with Quickload PCR mix (New England Biolabs) or KAPA PCR mix (KAPA Biosystems). In S63del mice, the MPZ^S63del^ transgene was identified with the following primers: 5’ CCTGGCCATTGTGGTTTAC 3’; 5’ GGTAGCGCCAGGTCAAGAT 3’. In C3 mice, identification of the transgene was performed according to The Jackson Laboratory’s genotyping protocol 21,197: Standard PCR Assay - Tg(PMP22)C3Fbas. The knockout of PMP22 was identified using the following primers: 5’ ATGCTCCTACTCTTGTTGGG 3’; 5’ AGATTAGCCACAGCCATAGTC 3’; 5’ GCCTTCTATCGCCTTCTTGAC 3’.

Biochemical experiments and morphological analyses were performed using sciatic nerves dissected from mice at the following ages: P30, P36-40 and P51-57. All behavioral and electrophysiology experiments were conducted during the light cycle on mice between the ages of P51-57.

Tadalafil (Selleckchem) and CYR-119 (provided by Cyclerion Therapeutics) were homogenously blended into OpenStandard Diet formula D11112201 by Research Diets, Inc. at a concentration of 100 mg/kg of chow, yielding an approximate daily dose of 10 mg/kg per mouse. Mice had *ad libitum* access to the chow throughout all studies. OpenStandard Diet formula D11112201 was given to the non-treatment groups and to all mice for 5 days prior to the start of treatment. The duration of the treatment was either 7 days (P29-P33 to P36-P40) or 21 days (P30-P36 to P51-P57). The mice were weighed every 3 days throughout each treatment.

### Nerve conduction studies

Sciatic nerve conduction velocity, F-wave latency, and distal/proximal onset latency of P51-57 mice were recorded following anesthetization with 0.4 mg/g of 2,2,2-tribromoethanol (Avertin-Sigma) in 1X PBS. Mice were placed under a heat lamp during all recordings to maintain core body temperature. The temperature of the table surface was monitored before and after the assessment of each mouse and kept between 36.5 and 38 °C. Steel monopolar needle electrodes were inserted as follows: recording lead within the muscle of the paw, reference lead between two digits, a ground lead in the cervical musculature, and two stimulating leads (cathode and anode) inserted along the sciatic nerve at the ankle, sciatic notch, and iliac crest. The distance between the stimulation sites was measured to calculate nerve conduction velocity (NCV). All measurements were obtained from right and left sciatic nerves using either a TECA Synergy N2 EMG stimulator or a Natus UltraPro S100 EMG/NCS/EP Neurodiagnostic System equipped with Synergy software.

### Rotarod

Motor coordination of P51-57 mice was assessed with a Ugo Basile Rotarod. Three 5-minute trials with acceleration from 4 to 40 rpm were performed in morning and afternoon sessions (≥ 6 h apart) over two consecutive days (four sessions total). The average latency to fall of the three trials was calculated for each mouse per session. Trial end was considered a fall from the rotarod or 3 consecutive passive rotations.

### Morphology

Sciatic nerves were dissected and submerged in 2% glutaraldehyde for a minimum of 24 h and then washed 3 times in phosphate buffer 0.12 M pH 7.4 (PB) at room temperature (RT). Nerves were then post-fixed at RT in 1% osmium tetroxide in PB for 2 h protected from light, washed 3 times in PB, and then sequentially dehydrated for 10 min at RT in ethanol of each of the following percentages: 50, 70, 90, 100, and 100. Next, nerves were washed twice in 100% propylene oxide for 10 min at RT and placed in a 1:1 mix of propylene oxide and epoxy resin overnight, with the vial open to air to allow the propylene oxide to evaporate. Nerves were embedded in epoxy resin and kept at 60 °C overnight to allow the resin to polymerize. The embedded nerves were then sectioned semithin (0.5 μm) and stained with 1% toluidine blue and 1% sodium borate for 5 min, or ultrathin (110 nm) and stained with 2% uranyl acetate in distilled water and Reynold’s lead citrate for 5 min. Stained semithin sections were then imaged at 100X on a Leica DM 6000B and ultrathin sections were imaged at 2900X on an FEI Tecnai G2 Spirit BioTWIN electron microscope (EM). These images were used for the morphological assessment of myelinated and dysmyelinated fibers, and to evaluate myelin thickness. For G-ratio analysis, at least 50 fibers from 15 EM images were measured per mouse. Unmyelinated fibers were analyzed on composite images of semithin sciatic nerve sections that were stitched together using PTGui software. Remak bundles were not counted as unmyelinated fibers in our analysis. At least 3,570 axons were quantified per nerve using Image J (NIH) software.

### Sciatic nerve lysis for immunoblotting

Snap-frozen sciatic nerves were pulverized in liquid nitrogen, suspended in Radioimmuno Precipitation Assay (RIPA) buffer (50 mM Tris-HCl pH 7.5, 150 mM NaCl, 1% NP-40, 0.5% sodium deoxycholate, 0.1% SDS, 5 mM EDTA, 1 mM NaF, 0.1 mM phenylmethylsulfonyl fluoride (PMSF), and 10 mM N-ethylmaleimide), sonicated 3 times at 65% power in a Bandelin Sonopuls HD 2200 for 20 s on − 30 s off, and centrifuged at 12,000 x g for 10 min at 4 °C. The supernatant was collected, protein concentration was determined using the Bicinchoninic acid assay (BCA, Thermo Fisher Scientific), and then samples were diluted to equal protein concentrations in RIPA buffer. 4X Laemmli Sample Buffer (Bio-Rad) was added to a final concentration of 1X, and samples were then boiled for 5 min at 95 °C. Equal volumes of each sample were loaded into Bis-Tris gels (GenScript) and separated via SDS-PAGE using MOPS (Tris-MOPS-SDS Running buffer powder, M00138 GenScript) or MES (NuPage MES 20X running buffer, NP0002 Thermo Fisher Scientific) running buffers. Gels were either wet transferred overnight at 4 °C in Tris-Bicine Transfer Buffer (24.8 mM Tris Base, 25 mM Bicine, 15% Methanol) or semi-dry transferred via a TransBlot Turbo system (Bio-Rad) to 0.45 μm PVDF (Immobilin FL, Millipore) or Nitrocellulose (Protran, Amersham) membranes.

For enzymatic deglycosylation, sciatic nerve lysates were prepared in RIPA buffer, as described above, and incubated with PNGase F (500 units/µL) or EndoH (500 units/µL) (New England Biolabs) under denaturing conditions according to the manufacturer’s protocol. The reaction was terminated by the addition of 4X Laemmli Sample Buffer (Bio-Rad) and incubation at 95 °C for 5 min. Equal amounts of protein from PNGase treated and non-treated samples were loaded in Bis-Tris gels and analyzed as described above.

The antibodies listed in the table below were used according to the manufacturer’s recommendations. To assess ubiquitin conjugates, nitrocellulose membranes were autoclaved after transfer. Protein bands were detected with the Odyssey CLx infrared imaging system (LiCor) and quantified with ImageJ (NIH) or ImageStudioLite (LiCor) software.

**Table:** 

Target (clone)	Host Species	Company	Catalog Number
Ubiquitin (P4D1)	Rabbit	Santa Cruz Biotechnology	sc-8017
Ubiquitin (VU-1)	Mouse	LifeSensors	VU101
K48-Ubiquitin	Rabbit	Cell Signaling	8081
PSMA6	Rabbit	Bethyl Laboratories	A303-809
PSMC2	Rabbit	Bethyl Laboratories	A303-822
GAPDH	Mouse	ProteinTech	60004-1-Ig
Beta Tubulin	Rabbit	Novus	NB600-936
Beta Tubulin	Mouse	Sigma-Aldrich	T4026
GAPDH	Rabbit	Sigma-Aldrich	G9545
GRP78	Rabbit	Novus	NBP1-06274
p-eIF2α	Rabbit	Cell Signaling	3398
eIF2α	Rabbit	Cell Signaling	9722
PMP22 (mouse)	Rabbit	Abcam	Ab303558
PMP22 (human)	Rabbit	Abcam	Ab270400

### Measuring proteasomal peptidase activity in sciatic nerve lysates

Sciatic nerves were pulverized in liquid nitrogen, suspended in APB (25 mM HEPES-KOH pH 7.5, 150 mM NaCl, 5 mM MgCl_2_, 1 mM ATP, 1 mM DTT and 10% glycerol) containing 0.1% NP-40, rotated end-over-end for 15 min at 4 ^°^C, sonicated (3 times, 10 s on − 30 s off, 25% power, Bandelin HD 2200), and centrifuged at 14,000 x g for 10 min at 4 ^°^C. The chymotrypsin-like peptidase activity of the proteasome was assessed in these sciatic nerve lysates as previously described [20].

### Measuring cGMP in sciatic nerve lysates

The concentration of cGMP in sciatic nerve lysates was measured using a cGMP ELISA (Cayman Chemical), as per manufacturer’s instructions. All solutions were made with ultrapure water (Cayman Chemical). Briefly, flash-frozen sciatic nerves were pulverized in liquid nitrogen, suspended in 0.1 N HCl, incubated for 20 min at RT while rotating end-over-end, and centrifuged at 1,000 x g for 10 min at RT. Samples were diluted 1:2 in the provided ELISA buffer and protein concentration was determined by BCA. All samples were measured in duplicate at two concentrations within the range of the standard curve. The concentration of cGMP was calculated using the cGMP non-acetylated ELISA program in My Assays (Cayman Chemical).

### Measuring the expression of sGC subunits

RNA was isolated from the sciatic nerves from P30 S63del mice, C3 mice, and WT littermates via Trizol (Invitrogen) extraction. Nerves were pulverized on liquid nitrogen and 400 µl of cold Trizol was added. Lysates were passed several times through a 26 5/8-gauge needle. 80 µL of chloroform was added and the lysates were then centrifuged at 12,000 x g for 15 min at 4^ °^C. 180 µL of the aqueous phase was combined with 200 µL of cold isopropanol, 1 µL glycogen (20 mg/µL), and 38 µL of sodium acetate (3 M). These mixtures were stored at -80 ^°^C for 1 h, to allow the RNA to precipitate, and then centrifuged at 12,000 x g for 10 min at 4 ^°^C. The supernatant was added to 1 mL of 75% cold ethanol and then centrifuged at 7,600 x g for 5 min at 4 ^°^C. This was repeated three times. The RNA pellet was then suspended in 10 µL of DEPC water and concentration and purity were analyzed on a Nanodrop 2000c (Thermo Fisher). 1 µg of RNA was reverse transcribed to cDNA using the SuperScript™ III First Strand Synthesis System (Invitrogen). Quantitative PCR was performed in a Bio-Rad CFX96/384 real time PCR detection system with Taqman probes (Thermo Fisher) specific for *guanylate cyclase 1 alpha 3 subunit* (Mm01220285_m1), *guanylate cyclase 1 beta 3 subunit* (Mm00516926_m1), and *beta-actin* (Mm01205647_g1). The data were calculated with the 2(−ΔΔCt) method.

### Immunohistochemistry on teased sciatic nerve fibers

The sciatic nerves from wild type and C3 mice, age P30, were fixed in cold 4% paraformaldehyde for 30 minutes and then washed 3 times in 1X phosphate buffered saline (PBS). Nerves were teased on 4% 3-aminopropyltriethoxysilane coated slides and then stored at -80 ^°^C. Nerves were permeabilized in cold methanol for 5 minutes, then washed 3 times in 1X PBS. A hydrophobic barrier was drawn around the nerve using a PAP pen (Vector Laboratories H-4000) and the nerves were blocked in a solution of 5% gelatin from cold water fish skin (Sigma G7041) and 0.5% Triton X-100 (Sigma T8787) in 1X PBS (blocking buffer) for 1 hour at room temperature. The primary antibodies were mouse anti KDEL (Enzo Life Sciences ADI SPA 827) (1:100), rabbit anti mouse PMP22 (Abcam 303558) (1:500), and rabbit anti human PMP22 (Abcam 270400) (1:250). The primary antibodies were diluted in 5% gelatin from cold water fish skin and 0.2% Triton X-100 in 1X PBS and incubated overnight at 4 ^°^C in a humidified chamber. Slides were then washed 3 times in 1X PBS and incubated with the secondary antibodies for 1 hour at room temperature, protected from light. The secondary antibodies were diluted in the blocking buffer and were Alexa Fluor 594 IgG2a goat anti mouse (Jackson Laboratories 115-585-206) (1:200) and Alexa Fluor 488 IgG (H + L) donkey anti rabbit (Jackson Laboratories 711-545-152) (1:400). The slides were then washed 3 times in 1X PBS and stained with 4’,6-diamidino-2-phenylindole (DAPI) (1:10,000) for 5 min. After 3 more washes in 1X PBS, the slides were air-dried and the PAP pen barrier was removed. The nerves were then covered with Vectashield antifade mounting medium (Vector Laboratories H-1000), followed by a glass coverslip. The slides were stored at -20 ^°^C in the dark until imaging. Images were taken with the 100X oil objective on a Leica TCS SP5 II confocal microscope.

### Statistics

The experimenter was blinded to genotype and treatment when performing behavioral, morphological, and functional experiments. For the analysis of F-wave latencies in C3 mice, we excluded any mouse from which an F-wave could not be measured from both the right and left sciatic nerves. GraphPad Prism 9.01 software was used for all statistical analyses. On all graphs, error bars are standard error of the mean (SEM) and * = *p* < .05; ** = *p* < .01; and *** = *p* < .001.

## Results

### Tadalafil and CYR119 increase proteasome peptidase activity and cGMP in peripheral nerves

**Fig. 1:**
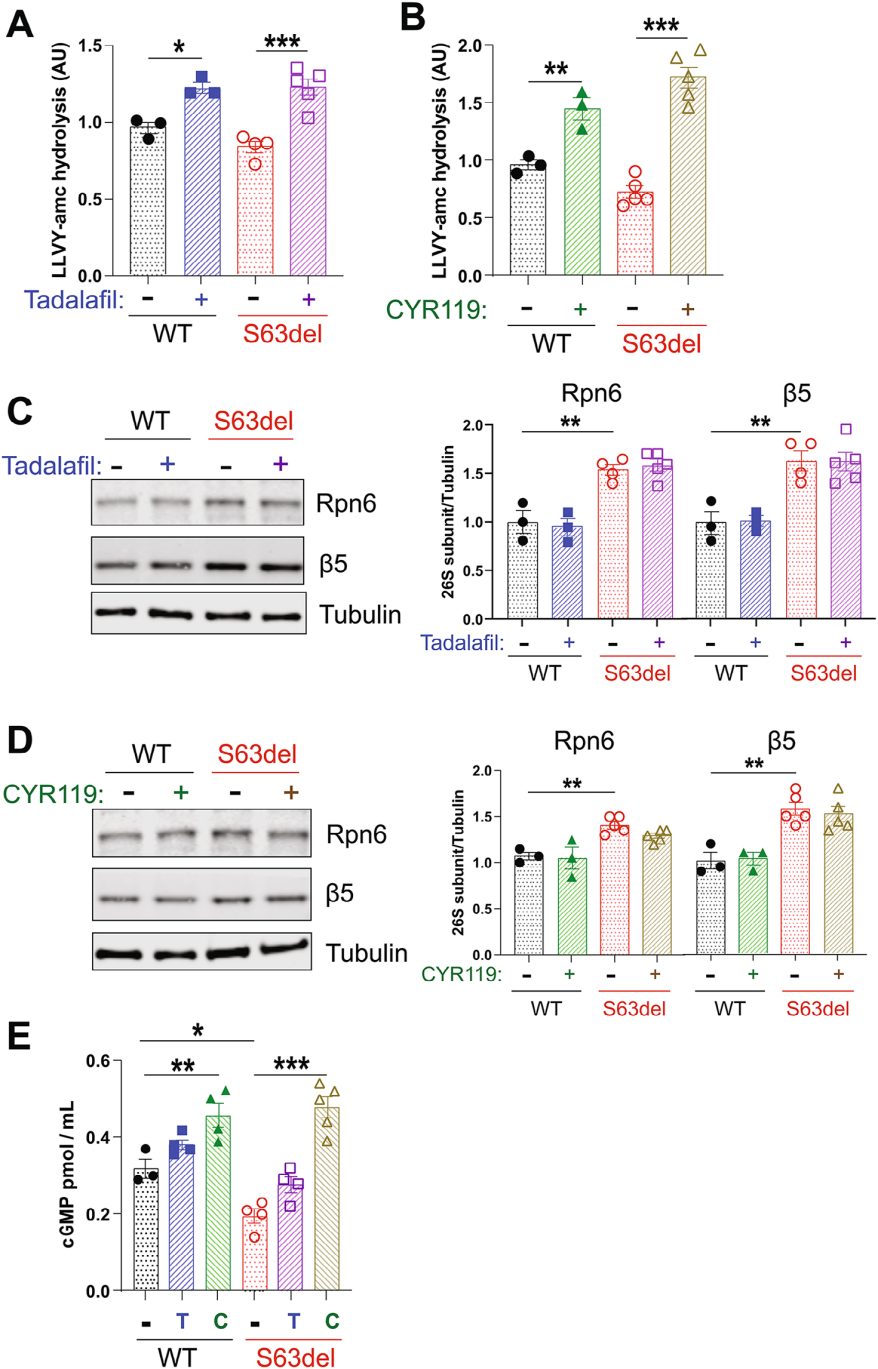
Tadalafil and CYR119 increase proteasome peptidase activities and cGMP in sciatic nerves of WT and S63del mice. **A**.) Tadalafil *per os* for 7 days increased the chymotrypsin-like activity of proteasomes in sciatic nerve lysates from WT and S63del mice. Here and below, *n* = 3–5 mice per genotype, per condition, and one-way ANOVA with a Bonferroni post-hoc analysis comparing WT and WT treated, WT and S63del, and S63del and S63del treated. The experiment was repeated with similar results. **B**.) CYR119 *per os* for 7 days increased proteasome chymotrypsin-like activity in sciatic nerve lysates from WT and S63del mice. **C**.) In sciatic nerve lysates from S63del mice, protein levels of the 26S proteasome subunits Rpn6 and β5 were increased. Tadalafil treatment for 7 days did not alter the levels of these subunits in WT or S63del. **D**.) CYR119 treatment for 7 days did not alter the levels of the 26S proteasome subunits Rpn6 or β5 in WT or S63del. **E**.) Tadalafil or CYR119 for 7 days increased the levels of cGMP in the sciatic nerve lysates, as measured by a cGMP ELISA. T indicates tadalafil and C indicates CYR119. Sciatic nerves from S63del mice had ~ 50% less cGMP than WT littermates

We previously showed that administration of the PDE5 inhibitor sildenafil via intraperitoneal injection to WT and S63del mice increased proteasome peptidase activities in the sciatic nerve lysates [20]. To determine whether the PDE5 inhibitor tadalafil has similar effects, S63del mice and WT littermates were treated with tadalafil for 7 days, beginning at P30, and then proteasomal peptidase activity was measured in the sciatic nerve lysates. Tadalafil was administered in the chow, to which the mice had *ad libitum* access. The tadalafil treatment increased proteasomal chymotrypsin-like activity by ~ 30% in WT and S63del mice (Fig. 1A). The proteasome’s caspase-like activity in the lysates was increased by a similar magnitude (Supplemental Fig. 1A). The coordinated increase in both peptidase activities strongly suggests that the proteasome activation involves more rapid entry of peptides into the 20S core, as seen previously with 26S proteasomes purified from human cells that had been treated with tadalafil [16], rather than stimulation of an individual 20S active site.

A second cohort of mice was treated with CYR119, a CNS-penetrant stimulator of the soluble guanylyl cyclase (sGC) [24], which was administered in the chow at the same dose as tadalafil. Sciatic nerve lysates from WT and S63del mice treated for 7 days with CYR119 exhibited a ~ 50% increase in proteasomal chymotrypsin-like activity (Fig. 1B) and caspase-like activity (Supplemental Fig. 1B).

Tadalafil and CYR119 both increased proteasomal peptidase activity in the lysates without altering the levels of 26S proteasome subunits, analyzed by western blot for the 19S subunit Rpn6 and the 20S subunit β5 (Fig. 1C and D). Therefore, the observed proteasome activation is likely due to the phosphorylation of pre-existent 26S proteasomes by PKG, as seen previously in several cultured human cell lines when PKG was activated by PDE5 inhibitors or sGC stimulators [16].

The lysates of sciatic nerves from untreated S63del mice contained higher levels of proteasome subunits than those from WT littermates (Fig. 1C and D), as reported previously [7, 20]. This increase in proteasomes is likely the reason why total proteasomal peptidase activities in the S63del lysates are similar to WT, because the peptide hydrolysis is normalized to total protein, not the number of proteasomes. Previous studies on 26S proteasomes purified from sciatic nerve lysates of S63del mice showed a decrease in specific activity [7]. It seems that Schwann cells in the sciatic nerves of S63del mice attempt to compensate for the decreased functionality of individual proteasomes by increasing the expression of proteasome subunits, as seen in many other cell types when proteasomes are inhibited pharmacologically [27]. However, more proteasomes do not fully compensate for the decrease in specific activity in sciatic nerves of S63del mice because polyubiquitinated proteins still accumulate (see below).

To verify that both compounds increased cGMP in the tissue of interest, we measured the concentration of cGMP in the sciatic nerve lysates of WT and S63del mice by ELISA. These experiments produced the unexpected finding that S63del sciatic nerve lysates had approximately half the amount of cGMP as WT (Fig. 1E). This deficiency in cGMP may be a previously unidentified contributor to the nerve pathology seen in the S63del mouse model of CMT1B. Tadalafil (T) increased cGMP levels by ~ 30% in both WT (0.32 pmol/mL to 0.38 pmol/mL) and S63del mice (0.2 pmol/mL to 0.28 pmol/mL) (Fig. 1E). CYR119 (C) increased the relative concentration of cGMP in both WT and S63del sciatic nerves to ~ 0.45 pmol/mL (Fig. 1E). In WT, this correlated to an increase of ~ 50%, and in S63del, to an increase of ~ 150%. The causes of the reduced levels of cGMP in S63del are unknown, but because CYR119, and not tadalafil, increased cGMP levels to the same relative concentration in WT and S63del, the deficiency in S63del is likely due to a reduction in the synthesis of cGMP, and not an increase in its breakdown. Analysis by qRTPCR of the levels of transcripts encoding the α and β subunits of sGC showed a ~ 25% decrease in the sciatic nerves from S63del mice (Supplemental Fig. 2A and 2B), suggesting that less expression of sGC could be one reason for the lower levels of cGMP. Combined, these experiments demonstrate that a 7-day treatment with tadalafil or CYR119 increases cGMP levels and proteasomal peptidase activity in the sciatic nerves of WT and S63del mice.

### Increasing cGMP in S63del mice for 21 days improves proteostasis

Because treatment for 7 days with CYR119 activated proteasome peptidase activities and increased the levels of cGMP in sciatic nerves of WT and S63del mice (Fig. 1), we extended the treatment to 21 days to evaluate its effects on proteostasis, myelination, and nerve conduction. Treatment began at P30, an age at which S63del mice already exhibit hypomyelination, slow electrical conduction, a UPR, and impaired protein degradation by the proteasomes in their sciatic nerves [6, 7]. We also included a tadalafil treatment group, enabling a comparison between tadalafil and CYR119 in these mice.

**Fig. 2:**
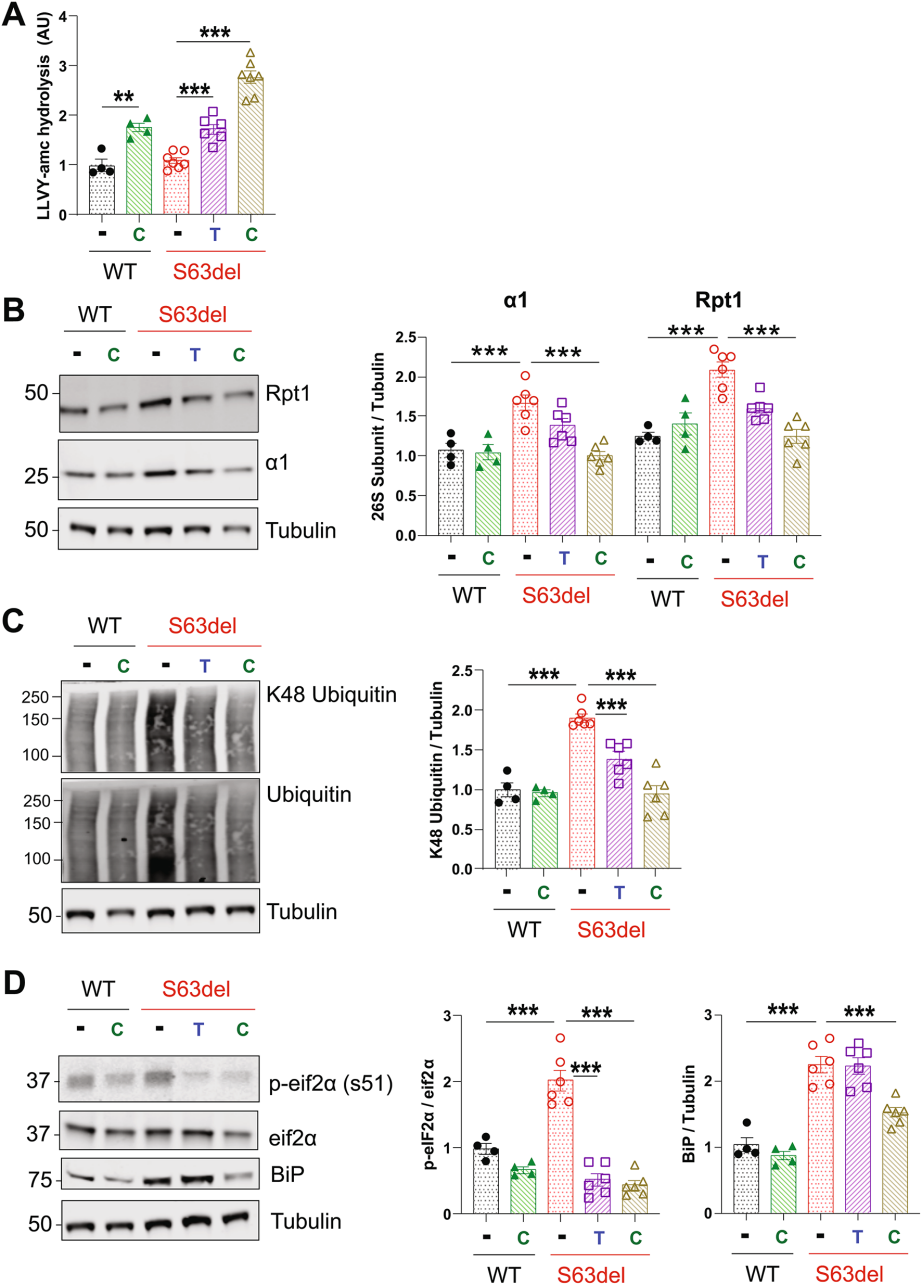
Treating S63del mice for 21 days with tadalafil or CYR119 improves proteostasis in the sciatic nerves **A**.) Raising cGMP with tadalafil (T) or CYR119 (C) increased proteasomal chymotrypsin-like activity in the sciatic nerve lysates of S63del mice. Here and below, *n* = 4–7 mice per genotype, per condition, and one-way ANOVA with a Bonferroni post-hoc analysis comparing WT and WT CYR, WT and S63del, S63del and S63del Tad, and S63del and S63del CYR. The experiment was repeated with similar results. **B**.) The levels of 26S proteasome subunits are higher in the sciatic nerves of S63del mice than in WT littermates. Tadalafil and CYR119 decreased the levels of the 20S subunit α1 and the 19S subunit Rpt1 in S63del mice, as determined by western blot. **C**.) S63del sciatic nerve lysates exhibit a 2-fold increase in polyubiquitinated proteins over WT littermates. Treatment with tadalafil or CYR119 reduced the levels of polyubiquitinated proteins. **D**.) Two indicators of proteotoxic stress, BiP and p-eif2α, are increased in the sciatic nerve lysates of S63del mice. Tadalafil and CYR119 reduced the levels of p-eiF2α. Only CYR119 reduced the protein levels of BiP

Treatment with CYR119 for 21 days increased proteasomal chymotrypsin-like activity by 2-3-fold in sciatic nerve lysates of WT and S63del mice (Fig. 2A). In WT mice, this treatment did not alter the protein levels of the 26S proteasome subunits Rpt1 and α1 in the sciatic nerve lysates (Fig. 2B). In untreated S63del mice, the levels of these subunits were higher than in WT, and the treatment with CYR119 reduced them to WT levels (Fig. 2B). Because the levels of 26S proteasome subunits decreased only in S63del, the treatment likely reduced the cause of the increased expression, which is, presumably, the accumulation of polyubiquitylated proteins.

Proteasomal protein degradation is reduced in the sciatic nerves of S63del mice [7], causing a 2-fold increase in polyubiquitinated proteins, including those linked through K48 which target proteins for degradation by 26S proteasomes (Fig. 2C). CYR119 treatment for 21 days reduced these ubiquitin conjugates to WT levels (Fig. 2C), presumably by increasing their degradation. The treatment did not alter the levels of polyubiquitinated proteins in WT mice (Fig. 2C).

In the sciatic nerves of S63del mice, MPZ^S63del^ accumulates in the ER of Schwann cells and induces a canonical UPR [5, 6]. Consequently, the levels of p-eif2α and the ER chaperone BiP are ~ 2-fold higher in the sciatic nerves of S63del mice than in WT littermates (Fig. 2D). The treatment with CYR119 reduced the levels of p-eif2α in S63del mice to levels below those seen in WT controls (Fig. 2D). The protein levels of BiP were also reduced (Fig. 2D). These decreases in markers of the UPR and in polyubiquitinated proteins strongly suggest that the CYR119 treatment ameliorated the proteotoxic stress observed in the sciatic nerves of S63del mice.

Tadalafil treatment of S63del mice for 21 days also increased proteasomal chymotrypsin-like activity (Fig. 2A) and reduced the levels of proteasome subunits (Fig. 2B), polyubiquitinated proteins (Fig. 2C), and p-eIF2α (Fig. 2D). In each case the magnitude of change with tadalafil was less than that observed with CYR119 - likely because tadalafil increased cGMP in sciatic nerves to a lesser extent than CYR119 (Fig. 1E). Tadalafil, like our previous treatments of S63del mice with sildenafil [20], did not reduce the levels of BiP (Fig. 2D), further demonstrating a greater reduction in proteotoxic stress with CYR119-mediated stimulation of sGC than that achieved with inhibition of PDE5.

### Treating S63del mice for 21 days with tadalafil or CYR119 restores myelin thickness in sciatic nerves

**Fig. 3:**
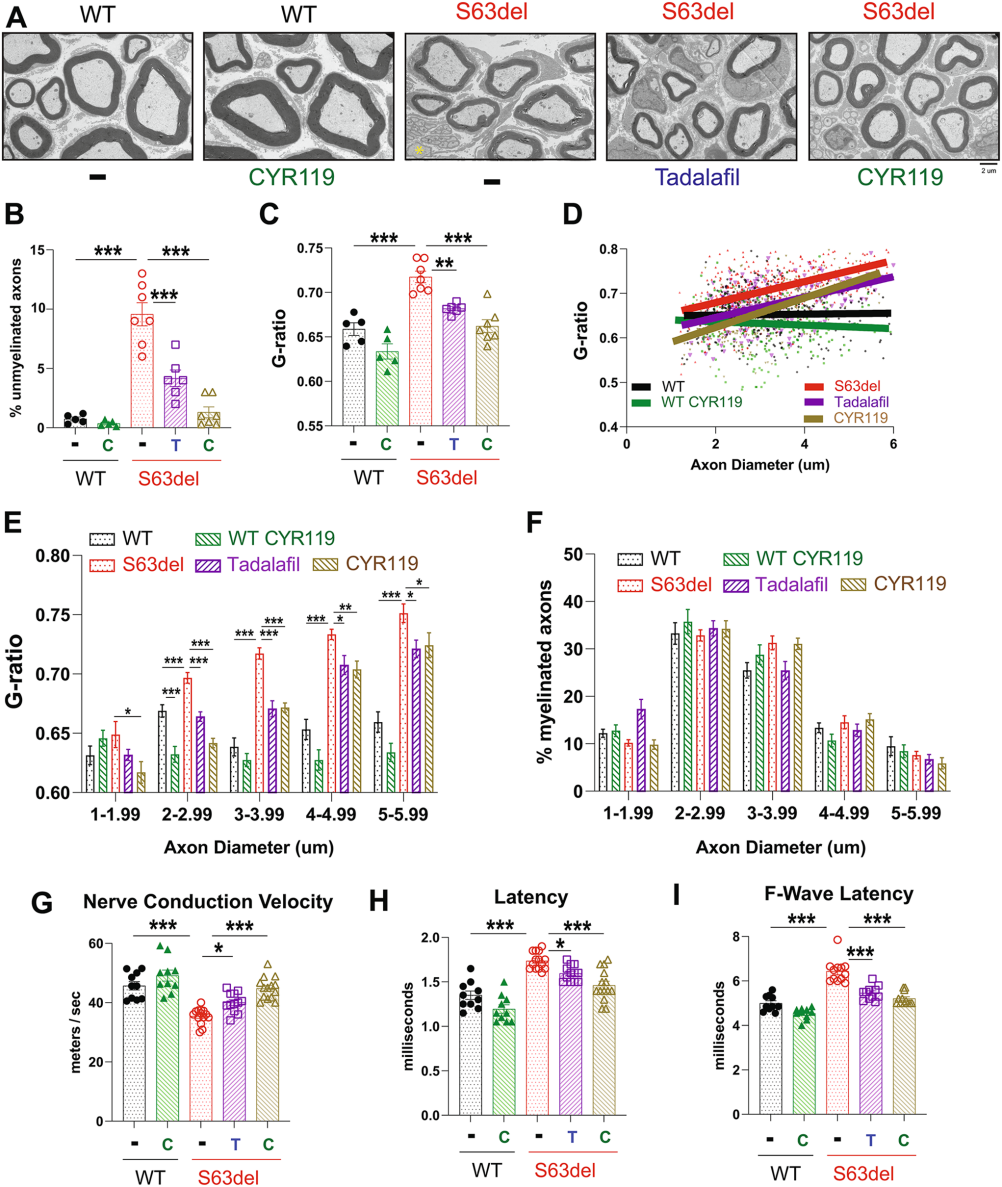
Treating S63del mice for 21 days with tadalafil or CYR119 restores myelin thickness and nerve conduction in sciatic nerves. **A**.) Representative electron microscopic images of ultrathin sections of sciatic nerves. The yellow asterisk indicates an unmyelinated fiber. **B**.) The % of unmyelinated fibers in sciatic nerves is higher in S63del mice than in WT littermates. The treatment with tadalafil (T) or CYR119 (C) reduced the number of unmyelinated fibers in the sciatic nerves of S63del mice. Here and below, *n* = 5–7 mice per genotype, per condition and one-way ANOVA with a Bonferroni post-hoc analysis comparing WT and WT CYR, WT and S63del, S63del and S63del Tad, and S63del and S63del CYR. **C**.) Average myelin thickness was lower in S63del sciatic nerves than in WT and was increased by tadalafil or CYR119. G-ratio = axon diameter / fiber diameter. Therefore, the higher the g-ratio, the thinner the myelin sheath. **D**.) A scatterplot of the g-ratio distribution across the measured axon diameters. S63del sciatic nerves have thinner myelin across all axon diameters, as indicated by the red line (S63del) being always above the black line (WT). Tadalafil and CYR119 treatments of S63del mice increased myelin thickness across all axon diameters, as indicated by the purple (tadalafil) and gold (CYR119) lines always being under the red line (S63del). **E**.) Graphs of the g-ratio distribution by axon diameter shown in D. S63del has thinner myelin (higher g-ratio) than WT across all axon diameters. Tadalafil or CYR119 treatments of S63del mice increased myelin thickness (lowered g-ratio) across all axon diameters. **F**.) The three-week treatment with tadalafil or CYR119 did not cause statistically significant changes in the percentage of myelinated axons of any diameter. **G**.) Treatment with tadalafil or CYR119 increased conduction velocity in the peripheral nerves of S63del mice. Here and below, *n* = 5–7 mice per genotype, per condition, corresponding to 10–14 measurements, one per sciatic nerve, and one-way ANOVA with a Bonferroni post-hoc analysis comparing WT and WT CYR, WT and S63del, S63del and S63del Tad, and S63del and S63del CYR. **H**.) Tadalafil or CYR119 treatment reduced the longer distal latencies in the peripheral nerves of S63del mice. **I**.) Treatment with tadalafil or CYR119 reduced the longer F-wave latencies in S63del mice

The pathological hallmarks of human patients with CMT1B [28], and S63del mice [25], are the presence of unmyelinated axons in peripheral nerves, and demyelination. Unmyelinated axons are those that meet the requirements for myelination – a diameter greater than 1 μm and are present in a one-to-one relationship with a Schwann cell – but are not myelinated. Such fibers are rare in the sciatic nerves of WT mice (< 1% of all axons) (Fig. 3A and B, Supplemental Fig. 3), but are more prevalent in S63del, where they constitute ~ 10% of all axons (Fig. 3A and B, Supplemental Fig. 3). Treatment of S63del mice with CYR119 for 3 weeks reduced the incidence of unmyelinated axons to ~ 1% (Fig. 3A and B, Supplemental Fig. 3), a frequency indistinguishable from WT.

S63del mice have thinner myelin sheaths around the axons in their peripheral nerves than WT littermates due to uniform demyelination [25]. To determine whether raising cGMP and reducing proteotoxic stress restored myelination, we quantified myelin thickness per axon by electron microscopic analysis of ultrathin sections of sciatic nerves (Fig. 3A). Myelin thickness is quantified as a g-ratio, which is defined as axon diameter divided by fiber diameter. Therefore, the higher the g-ratio, the thinner the myelin sheath surrounding the axon. In S63del mice, the 21-day treatment with CYR119 reduced the average g-ratio of all axons to the levels of untreated WT mice (Fig. 3C). This increase in myelin thickness in S63del was seen across all axon diameters (Fig. 3D and E). CYR119 treatment did not alter the percentage of myelinated axons in S63del or WT mice (Fig. 3F).

Treating S63del mice with tadalafil also reduced the incidence of unmyelinated fibers and increased myelin thickness across all axon diameters without altering the percentage of myelinated axons (Fig. 3B and F). The magnitude of these improvements was less than those elicited by CYR119.

### Treating S63del mice with tadalafil or CYR119 improves nerve conduction

We performed electromyography (EMG) to investigate whether the observed increases in myelin thickness improved nerve conduction. S63del mice have slow nerve conduction velocities, and prolonged distal and F-wave latencies due to uniform hypomyelination (Fig. 3G, 3, and I). Distal latency is the time required for a stimulus to travel from the dorsum of the vertebral column to muscles in the hind paw. F-wave latency is the time it takes a supramaximal electrical stimulus to travel antidromically from the ankle to the spinal cord and then orthodromically along the sciatic nerve to muscles in the hind paw. In S63del mice, the CYR119 treatment restored nerve conduction velocity (Fig. 3G), distal latency (Fig. 3H), and F-wave latency (Fig. 3I) to WT levels. The tadalafil treatment also improved these measures of nerve conduction, but to a lesser extent than the CYR119 treatment.

Taken together, these data demonstrate that pharmacologically raising cGMP in the S63del mouse model of CMT1B activates proteasomes and restores proteostasis, myelin thickness and nerve conduction.

### The C3 mouse model of CMT1A exhibits similar pathomechanisms to the S63del mouse model of CMT1B

**Fig. 4:**
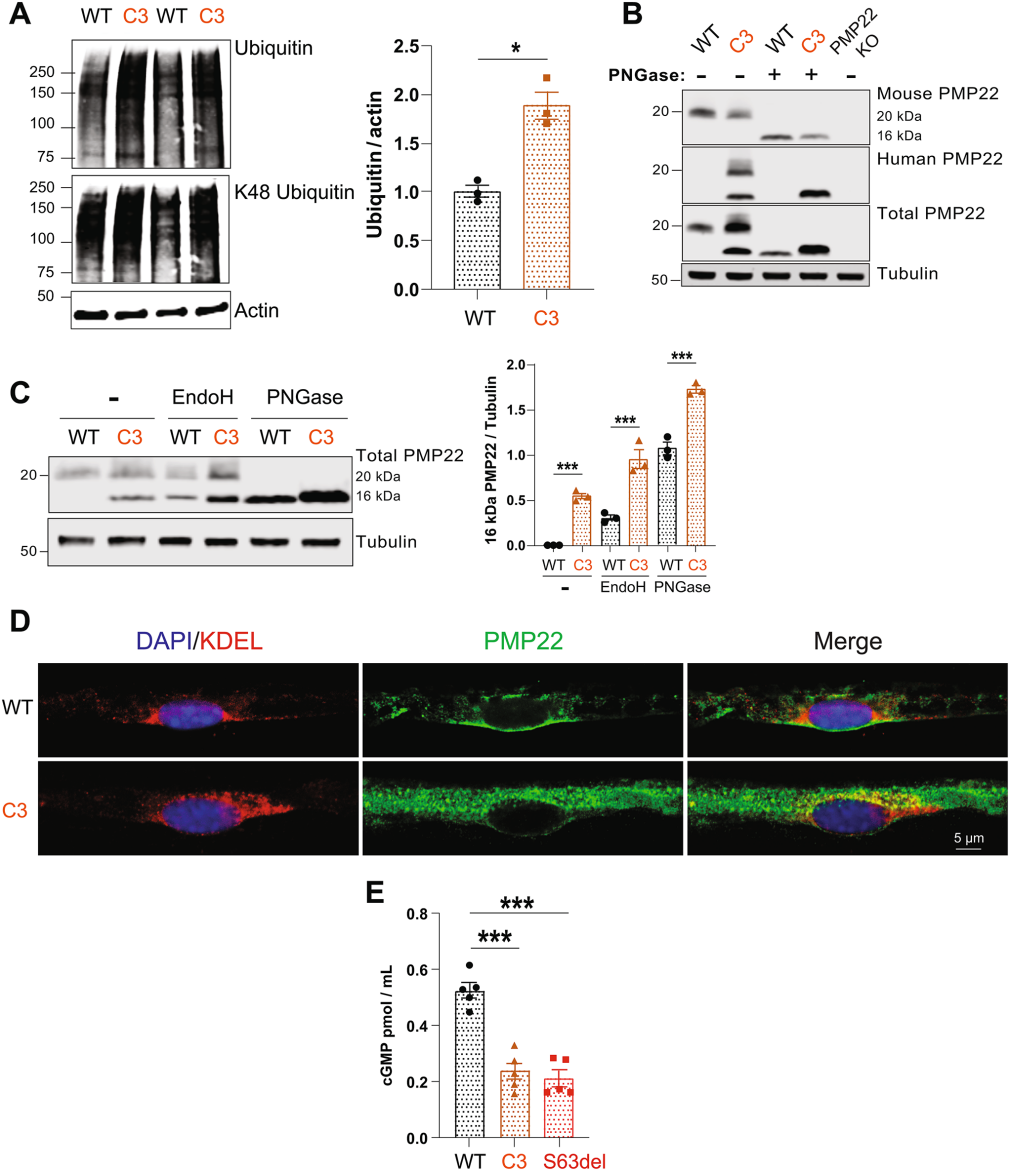
Polyubiquitinated proteins and PMP22 accumulate in the sciatic nerves of C3 mice. **A**.) The levels of polyubiquitinated proteins were higher in sciatic nerve lysates from C3 mice than from WT littermates. *n* = 3. Student’s t-test. **B**.) PMP22 accumulated in its glycosylated (20 kDa) and unglycosylated (16 kDa) forms in C3 sciatic nerves. Human PMP22 protein was detected only in lysates from C3 mice, as expected because C3 mice contain 3–4 copies of a human PMP22 transgene. Total PMP22 protein was detected by probing the blots simultaneously with one antibody specific for human PMP22 and a second antibody specific for mouse PMP22. Representative western blot of 3 experiments performed with lysates from 3 distinct mice per genotype. **C**.) EndoH-sensitive PMP22 is increased in the sciatic nerve lysates of C3 mice. The removal of all glycans in the lysates by PNGase showed that PMP22 is increased in the sciatic nerve lysates of C3 mice. *n* = 3 mice per genotype and one-way ANOVA with a Bonferroni post-hoc analysis comparing WT and C3. **D**.) PMP22 is localized in the ER of Schwann cells in sciatic nerves of C3 mice. Sciatic nerves from WT and C3 mice were teased into individual nerve fibers and immunohistochemical analysis was performed for KDEL, a marker of the ER, and human and mouse PMP22. The scale bar represents 5 μm. **E**.) The levels of cGMP were 50% lower in sciatic nerve lysates from C3 mice than from WT, as measured by cGMP ELISA. *n* = 5. One-way ANOVA with a Bonferroni post-hoc analysis comparing WT and C3, and WT and S63del. Experiment was repeated with similar results

Our promising results in the S63del mouse model of CMT1B prompted us to investigate whether compounds that increase cGMP could also treat the hereditary peripheral neuropathy CMT1A. CMT1A has a similar clinical presentation and pathological progression as CMT1B – demyelination of peripheral nerves that causes progressive distal weakness and muscle atrophy [2]. A clinically relevant model of CMT1A is C3 mice, which overexpress human PMP22 [29]. To determine whether CMT1A also has similar pathomechansims as CMT1B, and might therefore be treated by raising cGMP, we first performed western blot analysis for polyubiquitinated proteins. Sciatic nerve lysates from C3 mice contained approximately 2-fold more ubiquitin conjugates than WT littermates (Fig. 4A), suggesting a defect in their degradation by 26S proteasomes, as seen in the S63del mouse model of CMT1B (Fig. 2C).

To determine whether the disease-causing protein accumulates in the sciatic nerve lysates of C3 mice, a hallmark of proteotoxic diseases, we performed western blot analysis with two antibodies that detect PMP22 [30]. One antibody was specific for mouse PMP22 and detected a band at ~ 20 kDa, the observed molecular weight of PMP22, in both WT and C3 mice (Fig. 4B). C3 mice, in addition to their two copies of endogenous PMP22, harbor 3–4 copies of transgenic human PMP22 [29]. The second antibody we used was specific for human PMP22 and detected no PMP22 protein in lysates from WT mice (Fig. 4B), as expected. In sciatic nerve lysates from C3 mice, this antibody detected two bands, one at ~ 20 kDa and another at ~ 16 kDa (Fig. 4B), approximately the predicted molecular weight of PMP22. Enzymatic deglycosylation of the lysates with PNGase converted the 20 kDa band to the 16 kDa band (Fig. 4B), demonstrating that the 20 kDa band is glycosylated PMP22. The 16 kDa unglycosylated PMP22 was detected only in C3 mice in this study (Fig. 4B) and has been detected previously in the sciatic nerve lysates of C22 mice, a model which harbors 7 copies of human PMP22 and presents with a phenotype much more severe than that observed in patients with CMT1A [31], and in cultured rat Schwann cells in which glycosylation was inhibited by tunicamycin [32]. Probing the blots with both antibodies to simultaneously detect the endogenous and transgenic PMP22 revealed an increase in the levels of PMP22 protein in C3 sciatic nerves (Fig. 4B), shown even more clearly by enzymatic deglycosylation of the lysates with PNGase to convert PMP22 to one band (Fig. 4B and C). Thus, the level of PMP22 protein, especially pathological unglycosylated PMP22, is increased in the sciatic nerve lysates of C3 mice.

We also enzymatically deglycosylated the sciatic nerve lysates of WT and C3 mice with endoglycosidase (EndoH), which cleaves the high mannose N-linked glycans found on glycoproteins that have not fully trafficked through the Golgi. Sciatic nerve lysates from C3 mice contained approximately 3-times more EndoH-sensitive PMP22 than those from WT littermates (Fig. 4C). When PMP22 is incorporated into the myelin sheath, it contains mature glycans that are insensitive to hydrolysis by EndoH [33]. Therefore, in C3 mice, the levels of PMP22 are increased in the pre-Golgi space in the Schwann cells. PMP22 is a transmembrane protein. The pre-Golgi space in which it would most likely accumulate is the ER. We performed immunohistochemical analysis of teased sciatic nerve fibers from WT and C3 mice for KDEL, a marker of the ER, and PMP22. More PMP22 signal was detected in C3 mice than WT (Fig. 4D), as seen above by western blot analysis (Fig. 4B). In the WT nerves, there was little to no co-localization of KDEL and PMP22 (Fig. 4D), as expected because there was little EndoH-sensitive PMP22 in these nerves (Fig. 4C). In the nerves of C3 mice, KDEL and PMP22 co-localized, indicating that there is accumulation of PMP22 in the ER of Schwann cells in C3 mice (Fig. 4D).

Next, we measured the levels of cGMP in the sciatic nerve lysates of C3 mice and found a decrease of ~ 50% (Fig. 4E), similar to the magnitude of decrease seen in S63del mice (Figs. 1E and 4E). qRTPCR analysis found a 25–30% reduction in the levels of transcripts of the α and β sGC subunits in the sciatic nerves of C3 mice (Supplemental Fig. 4A and 4B), similar to the decreases seen in S63del (Supplemental Fig. 2A and 2B).

### A 7-day treatment of C3 mice with CYR119 increased proteasome activity and decreased polyubiquitinated proteins

**Fig. 5:**
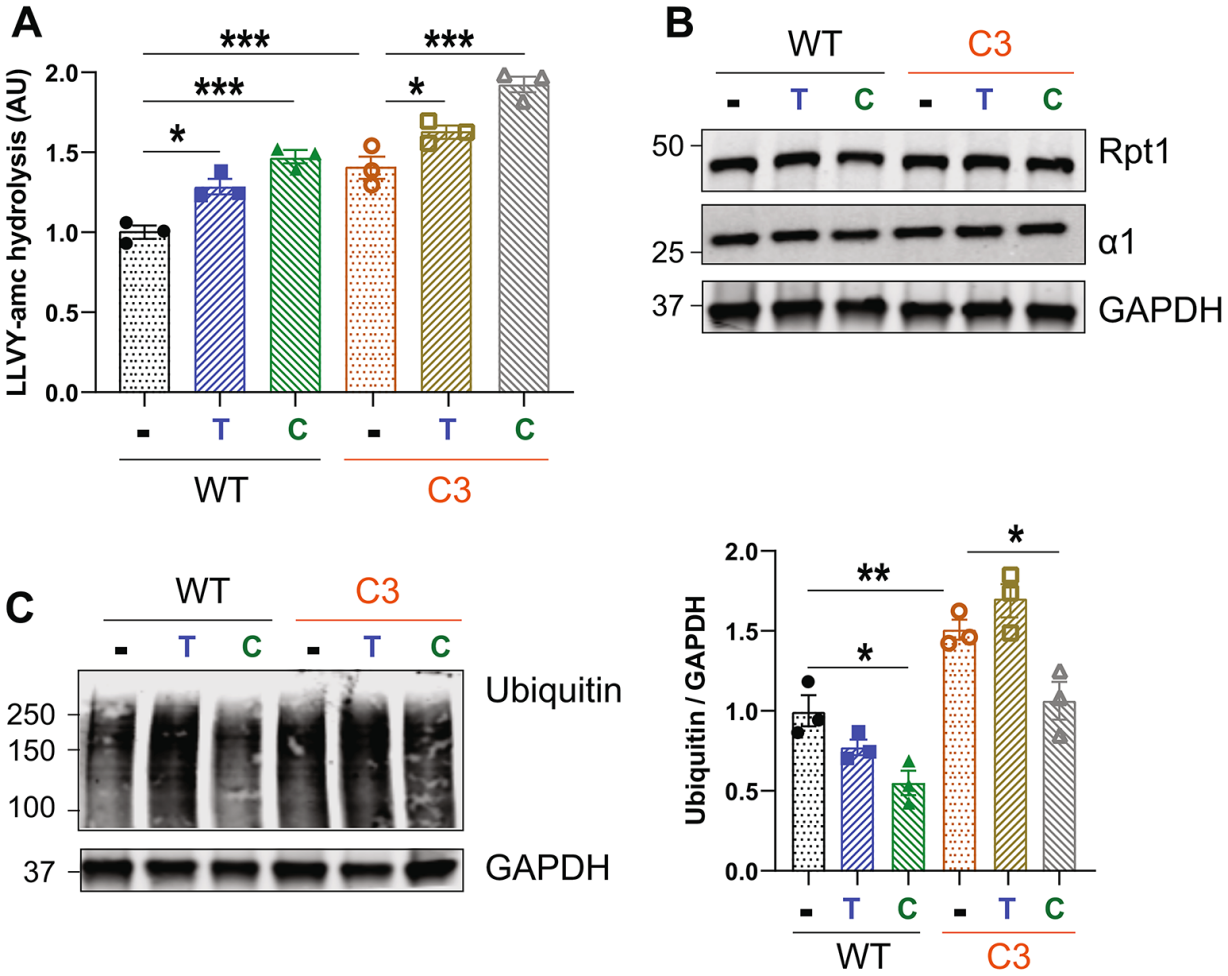
Treatment of C3 mice with CYR119 for 7 days increases proteasome peptidase activity in sciatic nerve lysates and reduces ubiquitin conjugates. **A**.) Treatment with tadalafil (T) or CYR119 (C) for 7 days increased proteasome chymotrypsin-like activity in sciatic nerve lysates of WT and C3 mice. Baseline proteasome peptidase activity was higher in C3 than in WT mice. Here and below, *n* = 3 mice per genotype, per condition and one-way ANOVA with a Bonferroni post-hoc analysis comparing WT and WT treated, WT and C3, and C3 and C3 treated. Experiment was repeated with similar results. **B**.) Levels of 26S proteasome subunits were similar in C3 mice and WT littermates. Neither treatment altered the protein levels of the 19S subunit Rpt1 or the 20S subunit α1. **C**.) CYR119 for 7 days reduced the levels of polyubiquitinated proteins in WT and C3 sciatic nerve lysates

Because C3 mice, like S63del mice, accumulate polyubiquitinated proteins (Fig. 4A) and the disease-causing protein in their sciatic nerves (Fig. 4B and C), and have decreased levels of cGMP (Fig. 4E), we sought to determine whether pharmacological agents that increase cGMP could also have therapeutic effects. First, we treated C3 and WT mice for 7 days with tadalafil or CYR119 and assessed proteasomal chymotrypsin-like activity in the sciatic nerve lysates. Both compounds increased this activity in WT mice (Fig. 5A) without changing the levels of proteasome subunits (Fig. 5B, Supplemental Fig. 5A and 5B), as seen above (Fig. 1A, B and C, and 1D). C3 sciatic nerve lysates exhibited proteasomal chymotrypsin-like activity that was ~ 40% higher than WT (Fig. 5A). This result was unexpected because polyubiquitinated proteins accumulated in the sciatic nerves of C3 mice (Fig. 4A), which often indicates a decrease in proteasomal degradation. To determine whether the increased proteasomal peptidase activity in C3 sciatic nerve lysates was caused by an increase in proteasomes, we performed western blot analysis for subunits of the 26S proteasome. The protein levels of the 19S subunit Rpt1 and the 20S subunit α1 in C3 sciatic nerve lysates were similar to that of WT (Fig. 5B, Supplemental Fig. 5A and 5B), showing that the higher peptide hydrolysis was not due to an increased level of proteasomes. Interestingly, C3 mice treated with tadalafil or CYR119 exhibited increased proteasomal peptidase activity in their sciatic nerves above the levels observed in untreated C3 mice (Fig. 5A).

The unexpected high levels of proteasomal peptidase activity in the sciatic nerve lysates of C3 mice prompted us to investigate whether further increasing this activity by pharmacologically raising cGMP could reduce the levels of polyubiquitinated proteins. Western blot analysis showed that the 7-day treatment of C3 mice with CYR119, but not tadalafil, reduced the levels of polyubiquitinated proteins towards levels seen in WT (Fig. 5C). Whatever the cause of the high proteasomal peptidase activity in C3 mice, this result suggests that the accumulation of polyubiquitinated proteins in C3 sciatic nerves is caused by a lack of degradation by 26S proteasomes, and that the treatment with CYR119 reduced the levels of polyubiquitinated proteins by increasing their degradation. We therefore chose to extend the treatment with CYR119 to 21-days to assess whether it could improve proteostasis, myelination, and nerve function in C3 mice.

### Treating C3 mice with CYR119 for 21 days improves proteostasis, myelination, and nerve conduction

**Fig. 6:**
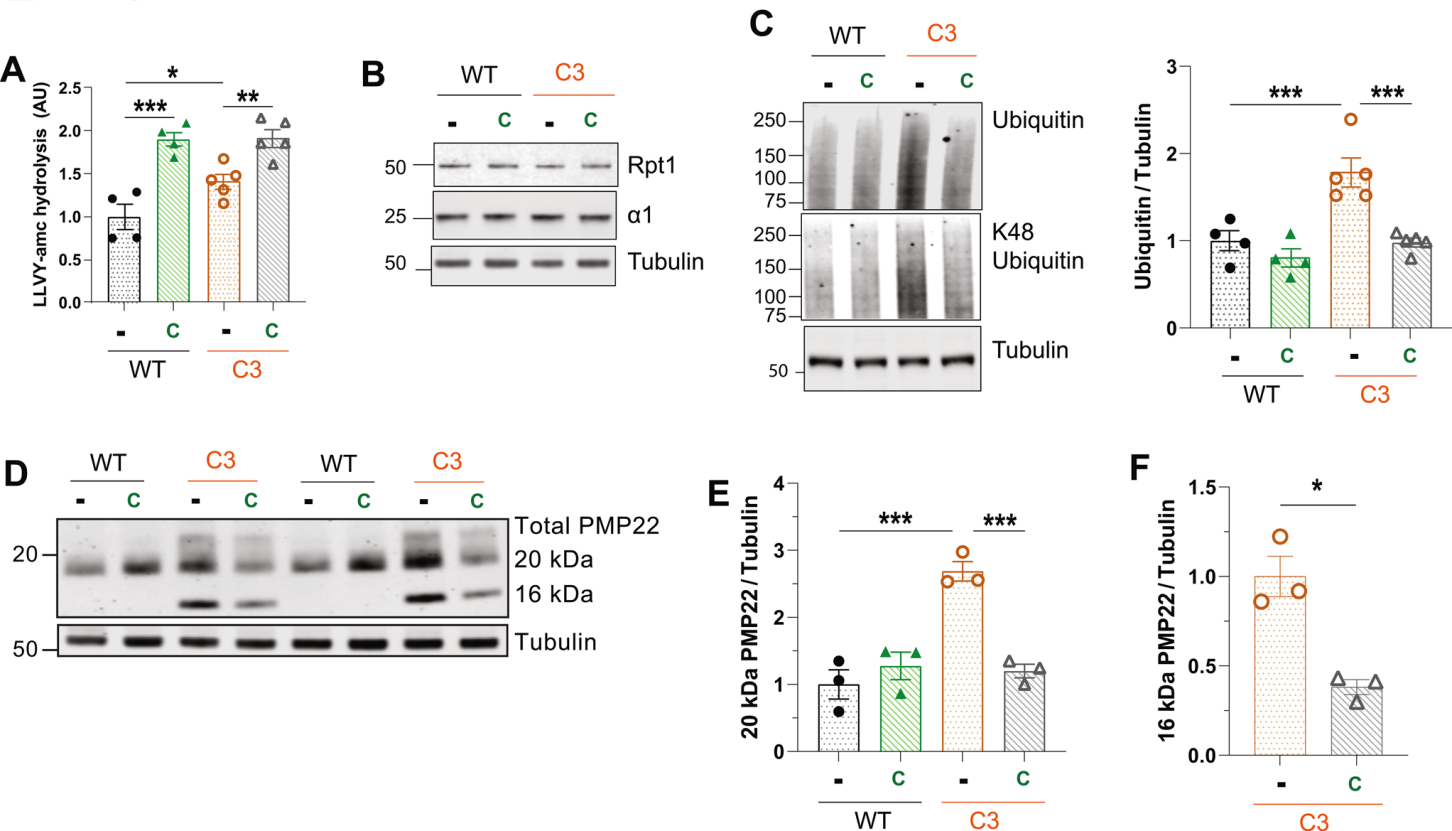
Treating C3 mice with CYR119 for 21 days increases proteasome peptidase activity and decreases the levels of PMP22. **A**.) CYR119 (C) for 21 days increased proteasome chymotrypsin-like activity in the sciatic nerve lysates of WT and C3 mice. Here and below, *n* = 4–5 mice per genotype, per condition and one-way ANOVA with a Bonferroni post-hoc analysis comparing WT and WT treated, WT and C3, and C3 and C3 treated. Experiment was repeated with similar results. **B**.) The levels of the 19S subunit Rpt1 and the 20S subunit α1 were similar in sciatic nerve lysates of WT and C3 mice and were unaltered by the treatment with CYR119. **C**.) Treating C3 mice for 3 weeks with CYR119 reduced the levels of polyubiquitinated proteins in the sciatic nerve lysates. **D**.) Glycosylated (20 kDa) and unglycosylated (16 kDa) PMP22 accumulated in the sciatic nerve lysates of C3 mice and their levels were reduced by the 3-week treatment with CYR119. **E**.) Treatment of C3 mice with CYR119 reduced the levels of glycosylated PMP22 (20 kDa). Here and below, *n* = 3 mice per genotype, per condition and one-way ANOVA with a Bonferroni post-hoc analysis comparing WT and WT treated, WT and C3, and C3 and C3 treated. **F**.) Unglycosylated PMP22 (16 kDa) was present only in C3 sciatic nerves and its levels were reduced by the treatment with CYR119. Student’s t-test

We treated a cohort of C3 and WT mice with CYR119 for 21 days and measured proteasomal chymotrypsin-like activity in the sciatic nerve lysates. C3 mice exhibited ~ 40% more of this activity than WT (Fig. 6A), as seen above (Fig. 5A). The treatment increased proteasomal chymotrypsin-like activity in both WT and C3 mice (Fig. 6A) without altering the levels of 26S proteasome subunits (Fig. 6B, Supplemental Fig. 6A and 6B). Western blot analysis for ubiquitin in the sciatic nerve lysates showed an approximately 2-fold higher level of polyubiquitinated proteins in C3 mice than in WT (Fig. 6C). The CYR119 treatment reduced the levels of polyubiquitinated proteins in C3 mice to those seen in WT (Fig. 6C). The sciatic nerve lysates were also analyzed for PMP22 by western blot with two antibodies to detect both the endogenous and transgenic PMP22, as performed above (Fig. 4B and C). In C3 mice, the CYR119 treatment reduced the ~ 20 kDa PMP22 to levels seen in WT and reduced the levels of the pathological ~ 16 kDa PMP22 by ~ 50% (Fig. 6D-F), presumably by increasing their degradation, as seen previously with MPZ^S63del^ [20].

**Fig. 7:**
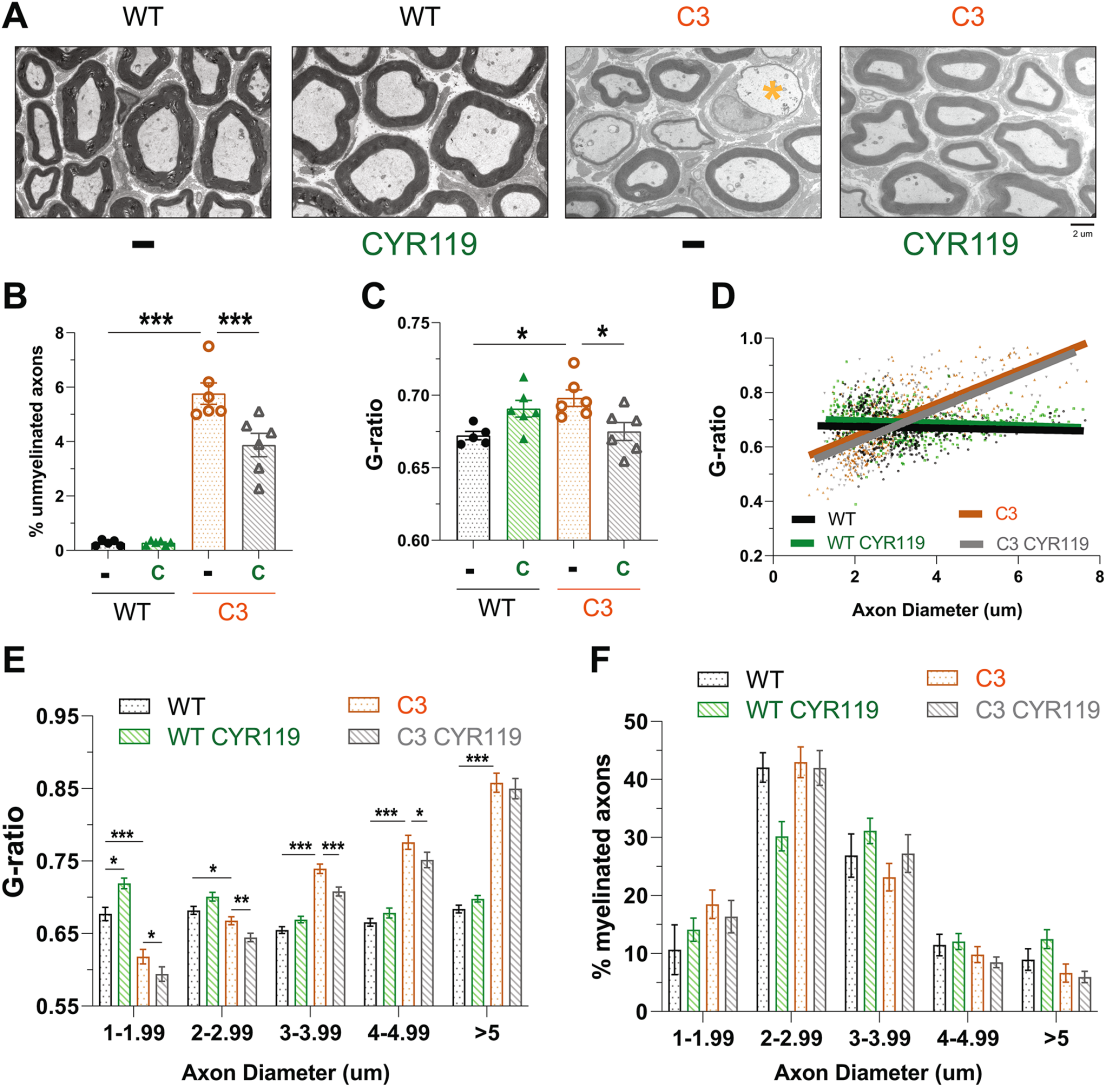
CYR119 treatment for 21 days improves myelination in the sciatic nerves of C3 mice. **A**.) Representative electron microscopic images of ultrathin sections of sciatic nerves. The yellow asterisk indicates an unmyelinated fiber. **B**.) C3 mice had more unmyelinated fibers in their sciatic nerves than WT littermates. The treatment with CYR119 (C) reduced the percentage of unmyelinated fibers. Here and below, *n* = 5–6 mice per genotype, per condition and one-way ANOVA with a Bonferroni post-hoc analysis comparing WT and WT treated, WT and C3, and C3 and C3 treated. **C**.) Average myelin thickness was lower (higher g-ratio) in C3 sciatic nerves and was increased (lower g-ratio) by the treatment with CYR119. **D**.) Scatterplot of the g-ratio distribution across the measured axon diameters. C3 sciatic nerves had thinner myelin (higher g-ratio) than WT around axons with diameters > 2 μm and thicker myelin (lower g ratio) around axons with diameters < 2 μm. **E**.) Bar graphs of the g-ratio distribution by axon diameter shown in D. CYR119 treatment of C3 mice increased myelin thickness across all axon diameters < 5 μm. **F**.) The three-week treatment with CYR119 did not cause statistically significant changes in the percentage of myelinated axons of any diameter in C3 mice

To assess whether raising cGMP was also improving the morphological and functional deficits associated with CMT1A neuropathy, we first analyzed images of semithin sections of sciatic nerves from WT and C3 mice for the presence of unmyelinated axons. In the sciatic nerves of C3 mice, unmyelinated axons constituted ~ 6% of all axons (Fig. 7B and Supplemental Fig. 7). The treatment with CYR119 reduced their incidence to ~ 4% (Fig. 7B and Supplemental Fig. 7).

C3 mice have a higher average g-ratio (i.e., thinner myelin sheaths) in their sciatic nerves than WT littermates (Fig. 7C) due to demyelination and dysmyelination [29]. However, in C3 mice, not all axons in the peripheral nerves are hypomyelinated, as they are in S63del mice (Fig. 3D and E). Instead, only the large caliber axons (> 3 μm in diameter) are hypomyelinated (i.e., higher g-ratio than WT), and the small caliber axons (< 3 μm in diameter) are hypermyelinated (i.e., lower g-ratio than WT) (Fig. 7D and E). Treatment of C3 mice for 21 days with CYR119 reduced the average g-ratio (Fig. 7C). This small but significant increase in myelin thickness was seen across axons < 5 μm in diameter (Fig. 7D and E). The CYR119 treatment did not alter the percentage of axons myelinated in C3 mice (Fig. 7F).

**Fig. 8:**
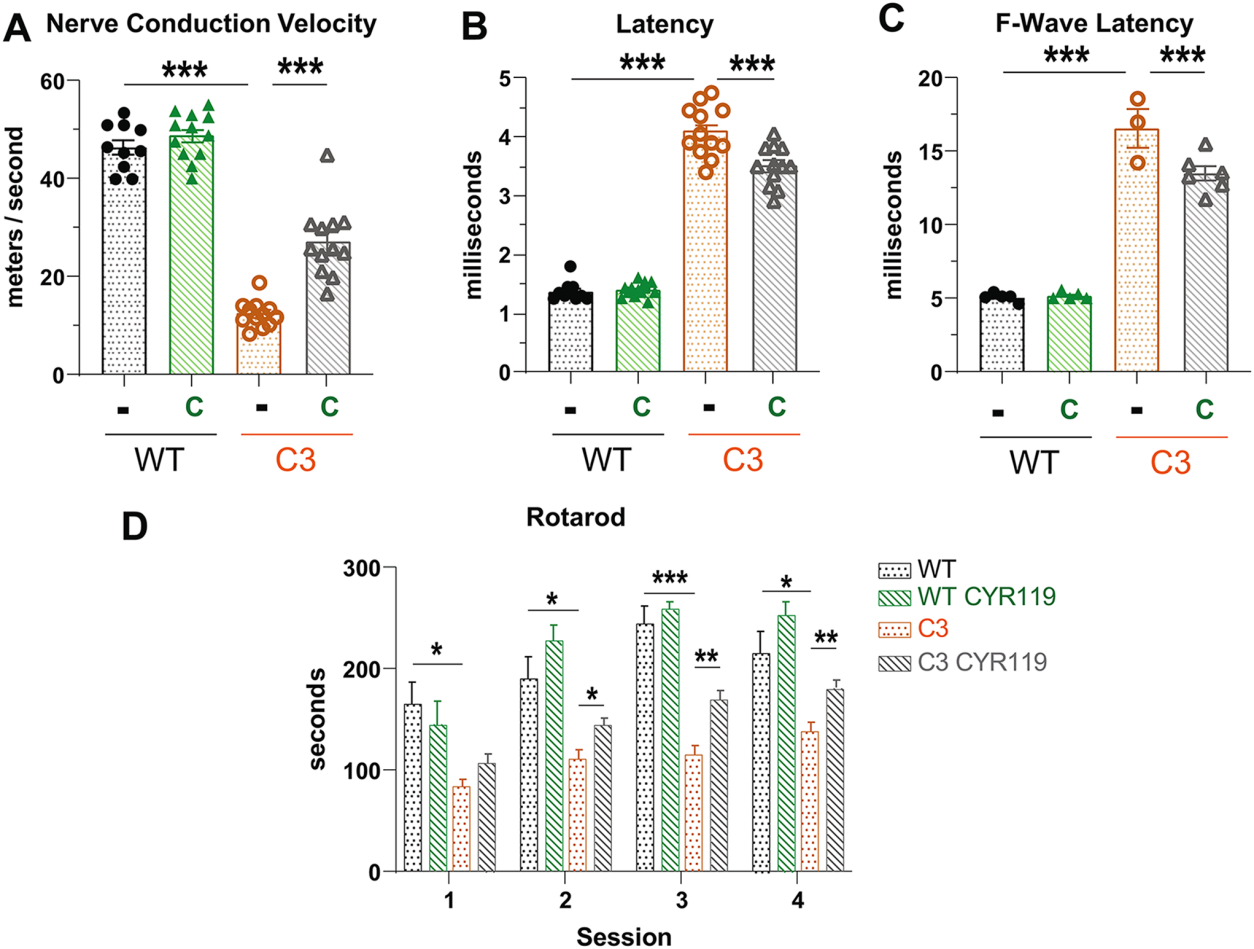
CYR119 treatment for 21 days increases nerve conduction and motor coordination in C3 mice. **A**.) Treatment with CYR119 (C) increased conduction velocity in the sciatic nerves of C3 mice. Here and below, *n* = 5–6 mice per genotype, per condition, corresponding to 10–12 measurements, one per sciatic nerve per mouse and one-way ANOVA with a Bonferroni post-hoc analysis comparing WT and WT treated, WT and C3, and C3 and C3 treated. **B**.) CYR119 treatment reduced the longer distal latencies seen in C3 mice. **C**.) Treatment with CYR119 reduced the longer F-wave latencies in C3 mice. *n* = 3–6 mice per genotype, per condition. Any mouse from which F-wave latencies could not be measured from both left and right sciatic nerves was excluded from the analysis. **D**.) On an accelerating rotarod, C3 mice fell off sooner than WT littermates. C3 mice treated with CYR119 ran longer than C3 controls in 3 out of the 4 sessions. *n* = 10–14 mice per genotype, per condition and one-way ANOVA with a Bonferroni post-hoc analysis comparing WT and WT treated, WT and C3, and C3 and C3 treated

To determine whether the decrease in incidence of unmyelinated fibers and the small increases in myelin thickness translated into improved function, we performed EMG studies to assess nerve conduction, and behavioral studies to assess motor coordination. C3 mice, like patients with CMT1A, exhibit slow nerve conduction velocities (Fig. 8A), and longer distal (Fig. 8B) and F-wave latencies (Fig. 8C). The treatment of C3 mice with CYR119 increased nerve conduction velocities (Fig. 8A) and reduced the distal (Fig. 8B) and F-wave latencies (Fig. 8C).

Motor coordination of WT and C3 mice was assessed by measuring the amount of time the mice could run on an accelerating rotarod without falling. C3 mice have significant deficits in motor coordination and fell from the rotarod quicker than their WT littermates across all 4 sessions (Fig. 8D). C3 mice treated with CYR119 were able to remain on the rotarod longer than untreated C3 mice in the final 3 trials (Fig. 8D), demonstrating an improvement in motor coordination. Thus, increasing cGMP levels in the C3 mouse model of CMT1A via *per os* treatment with CYR119 restored protein homeostasis, increased myelin thickness, and improved nerve conduction and motor function.

## Discussion

CMT1A and CMT1B are proteotoxic neuropathies for which there are no treatments. We previously reported that treating the S63del mouse model of CMT1B with the PDE5 inhibitor sildenafil restored proteostasis and lessened neuropathy [20], presumably by reducing the accumulation of MPZ^S63del^ in Schwann cells by enhancing its degradation by 26S proteasomes. In this present study, we further tested whether compounds that increase cGMP could be viable treatments for CMT1 neuropathies and expanded upon our previous results in several ways. We altered the route of administration from intraperitoneal injections to *per os*, to better model in mice how these molecules would be used in a therapeutic setting by patients. We tested two classes of compounds that increased cGMP via the NO pathway: the PDE5 inhibitor tadalafil and the brain-penetrant sGC-stimulator CYR119. Treating CMT1 does not necessarily require a molecule that enters the CNS, but generally, molecules that cross the blood brain barrier also cross the blood nerve barrier. Some molecules, such as sildenafil, do not enter the brain, but do enter the nerves [20]. Further examination is required to determine if non-brain penetrant sGC stimulators [34] enter the peripheral nerves and could potentially treat CMT1 neuropathies. We also treated the C3 mouse model of CMT1A with CYR119 to test whether the positive effects caused by pharmacologically increasing cGMP are seen in other demyelinating neuropathies.

Raising cGMP in cells by stimulating its synthesis by sGC or inhibiting its breakdown by PDE5 enhances protein degradation by the UPS [16, 20]. The 26S proteasome is directly phosphorylated by PKG on a not-yet-identified subunit or interacting protein, and because of this phosphorylation, it more rapidly hydrolyzes its physiological substrates: ATP and polyubiquitinated proteins [16]. Activating PKG also increases protein ubiquitination [16, 35]. It is likely that both the increased ubiquitination and proteasome activation contribute to the observed stimulation of intracellular protein breakdown, and reduction of proteotoxicity in models of CMT1, however, this has yet to be tested. Such simultaneous increases in ubiquitination and proteasomal function have also been seen during heat shock of mammalian cells [36] and may also occur with other kinases reported to activate the proteasome via subunit phosphorylation [12]. It is possible that the proteins that are more ubiquitinated when cGMP levels increase are the same proteins that are degraded by the activated 26S proteasomes. When MPZ^S63del^ was expressed in HEK293 cells, raising cGMP increased both its ubiquitination and degradation. MPZ^WT^ was not ubiquitinated more when cGMP was raised [20], and its rate of degradation was unaltered, supporting the idea that increased ubiquitination of a protein correlates with increased degradation when 26S proteasomes are activated by phosphorylation. Further experiments are required to determine whether these observations are generalizable to the other mutant, disease-causing proteins that are degraded more when cGMP levels are increased (e.g., tau, αβ crystallin, and huntingtin) [16, 18].

The treatments of S63del mice with CYR119 or tadalafil reduced the intracellular accumulation of misfolded proteins and abrogated the negative morphological and functional consequences. In the Schwann cells of S63del mice, MPZ^S63del^ accumulates in the endoplasmic reticulum [6], where its continued presence elicits several markers of proteotoxic stress. One is the UPR, which upregulates molecular chaperones and enzymes involved in the ERAD pathway and activates PERK to phosphorylate eif2α. These actions are generally adaptive and attempt to lessen the cellular burden of misfolded proteins by increasing folding capacity and degradation of misfolded proteins, and by reducing protein synthesis, respectively [37]. In S63del mice, the genetic ablation of the ERAD enzyme Derlin2 [38] or the phosphorylation of eif2α [39] worsened neuropathy, indicating that these aspects of the UPR are beneficial in CMT1B. Prolonged activation of the UPR can be detrimental. In S63del mice, CHOP [5] and its downstream effector GADD34 [40, 41] cause demyelination of axons in motor nerves. Both tadalafil and CYR119 decreased markers of the UPR, most likely indicating less accumulation of MPZ^S63del^, and perhaps other proteins.

Another indicator of proteotoxicity in the peripheral nerves of S63del mice is an increase in the levels of proteasome subunits. A decrease in proteasomal protein degradation elicits the upregulation of all proteasome subunits via the transcription factor Nrf1. This transcriptional response has been well-described with pharmacological inhibition of proteasomes in mammalian and plant cells [27], and is also observed in S63del mice [7]. The cause of the proteasomal impairment in S63del mice is not clear, but one possibility is the binding of MPZ^S63del^ to 26S proteasomes, as has been documented with other mutant proteins that accumulate in the affected cells and cause disease [42]. Tadalafil and CYR119 reduced the levels of proteasome subunits, most likely by decreasing the cause of the proteasome impairment: the continued presence of MPZ^S63del^.

In the C3 mouse model of CMT1A, the disruption of proteostasis and the cause(s) of demyelination are less well described than in S63del mice. C3 mice exhibit a slight UPR in their peripheral nerves, and a pharmacological treatment of these mice for 12 weeks to inhibit dephosphorylation of eif2α improved myelination, nerve conduction, and motor coordination, indicating some level of proteotoxicity in the Schwann cells of C3 mice, likely caused by the overproduction and accumulation of PMP22 [43]. Pharmacologically inhibiting GADD34’s dephosphorylation of eif2α is also beneficial in mouse models of CMT1B caused by the expression of distinct MPZ mutants (e.g., MPZ^S63del^ and MPZ^R98C^) [40, 43], suggesting shared pathomechanisms with CMT1A. We showed here that polyubiquitinated proteins accumulated in the affected peripheral nerves of C3 mice, as is also observed in S63del mice. From these observations, we expected to find the other markers of proteasome impairment. However, the levels of proteasome subunits were not increased in the sciatic nerves of C3 mice, and proteasomal peptidase activity in the lysates was greater than that measured in WT littermates. The reasons for this apparent disconnect between proteasome peptidase activity in the lysates and levels of polyubiquitinated proteins are not clear. Proteasomal peptide hydrolysis often correlates with the rate of hydrolysis of polyubiquitinated proteins, but not always. It is possible that in sciatic nerves of C3 mice, the proteasomes are active and competent for the hydrolysis of peptides, but not proteins, as was seen previously with the ubiquitination of proteasome subunits that bind polyubiquitinated proteins [44]. Whatever the cause of the unexpected results, importantly, the CYR119 treatment decreased the levels of polyubiquitinated proteins, indicating an improvement in protein homeostasis.

In this and prior studies, we were unable to show accumulation of MPZ^S63del^ in the sciatic nerves of S63del mice because of the massive amounts of MPZ^WT^ protein. PMP22 is far less abundant than MPZ, constituting ~ 5% of total protein compared to ~ 50% [3]. In C3 mice, we could therefore show an increase in its protein level directly. In the sciatic nerves of C3 mice, there was an increase in immature glycosylated and unglycosylated PMP22. These two forms are not found in the myelin sheath and are accumulating within the Schwann cells. Unglycosylated PMP22 is detected only in C3 mice and is associated with pathology. Treating C3 mice with CYR119 decreased the levels of PMP22 - glycosylated and unglycosylated, likely by increasing their degradation. We assume that it is through this mechanism that CYR119 improved biochemical, morphological, and neurophysiological markers of CMT1A neuropathy. However, because raising cGMP levels also causes vasodilation and increased blood flow to tissues [21], these treatments may have had other therapeutic benefits in vivo, in addition to enhancing the clearance of toxic proteins in the affected cells.

One unexpected finding in this study was that the levels of cGMP in the sciatic nerves of S63del and C3 mice were approximately 50% less than WT. Although the observed decrease in expression of sGC subunits is one mechanism through which cGMP synthesis could be reduced, the regulation of intracellular levels of cGMP is complex, and other possible explanations could include the cell-autonomous and non-cell autonomous production of NO, the redox state of sGC, and the expression and activity of phosphodiesterases [21]. Further study is required to determine which of these factors contribute to the decrease in cGMP observed in the sciatic nerves of S63del and C3 mice and whether there is less cGMP in the affected tissues in other models of CMT1, as well as other proteotoxic diseases.

The phenotypes of S63del and C3 mice were ameliorated by compounds that increased the levels of cGMP in sciatic nerves, raising the possibility that CMT1 could be considered a cGMPopathy [45]. Thus far, such disorders have been characterized in the cardiovascular system, where the functions of cGMP are well-described. Very little is known about the role of cGMP in Schwann cells and in myelination. More work is required to understand whether a decrease in cGMP could cause the failures of protein homeostasis and the decreases in myelin thickness observed in CMT1.

## Electronic supplementary material

Below is the link to the electronic supplementary material.

Supplementary Material 1

## Data Availability

All data is available from the corresponding author upon reasonable request.

## Declarations

### Disclaimers

The opinions and assertions expressed herein are those of the authors and do not reflect the official policy or position of the Uniformed Services University of the Health Sciences or the Department of Defense.
