# Supplementary material for: Pharmacologically increasing cGMP improves proteostasis and reduces neuropathy in mouse models of CMT1
Source: Cell Mol Life Sci. 2024 Oct 14;81(1):434. doi: 10.1007/s00018-024-05463-1 (PMC11473742; doi:10.1007/s00018-024-05463-1)
Supplement: Supplementary file 1 — Supplementary Material 1 [file 18_2024_5463_MOESM1_ESM.pdf]

Supplemental Figure 1

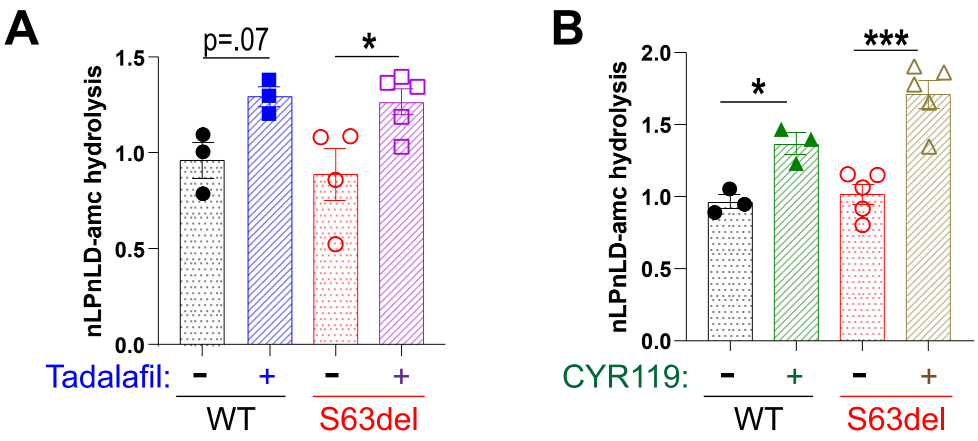

Supplemental Figure 1: Proteasomal caspase-like activity in WT and S63del sciatic nerve lysates

A.) Tadalafil for 7 days increased proteasomal caspase-like activity in sciatic nerve lysates from S63del mice and WT littermates. Here and below, n=3-5 mice per genotype, per condition, and one-way ANOVA with a Bonferroni post-hoc analysis comparing WT and WT treated, WT and S63del, and S63del and S63del treated. The experiment was repeated with similar results.

B.) CYR119 for 7 days increased proteasomal caspase-like activity in sciatic nerve lysates from S63del mice and WT littermates.

Supplemental Figure 2

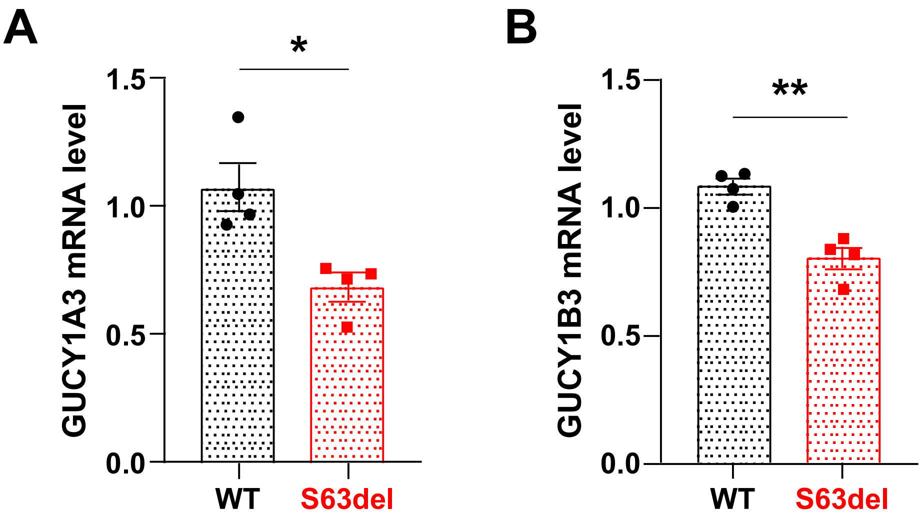

Supplemental Figure 2: Expression of soluble guanylyl cyclase (sGC) subunits is decreased in the sciatic nerves of S63del mice

A.) mRNA levels of an α subunit of sGC were decreased in the sciatic nerves of S63del mice. Here and below, n=4 mice per genotype. Student's t-test. Data were normalized to beta actin and calculated using the $2^{(-\Delta\Delta Ct)}$ method.

B.) mRNA levels of the β subunit of sGC were decreased in the sciatic nerves of S63del mice.

Supplemental Figure 3

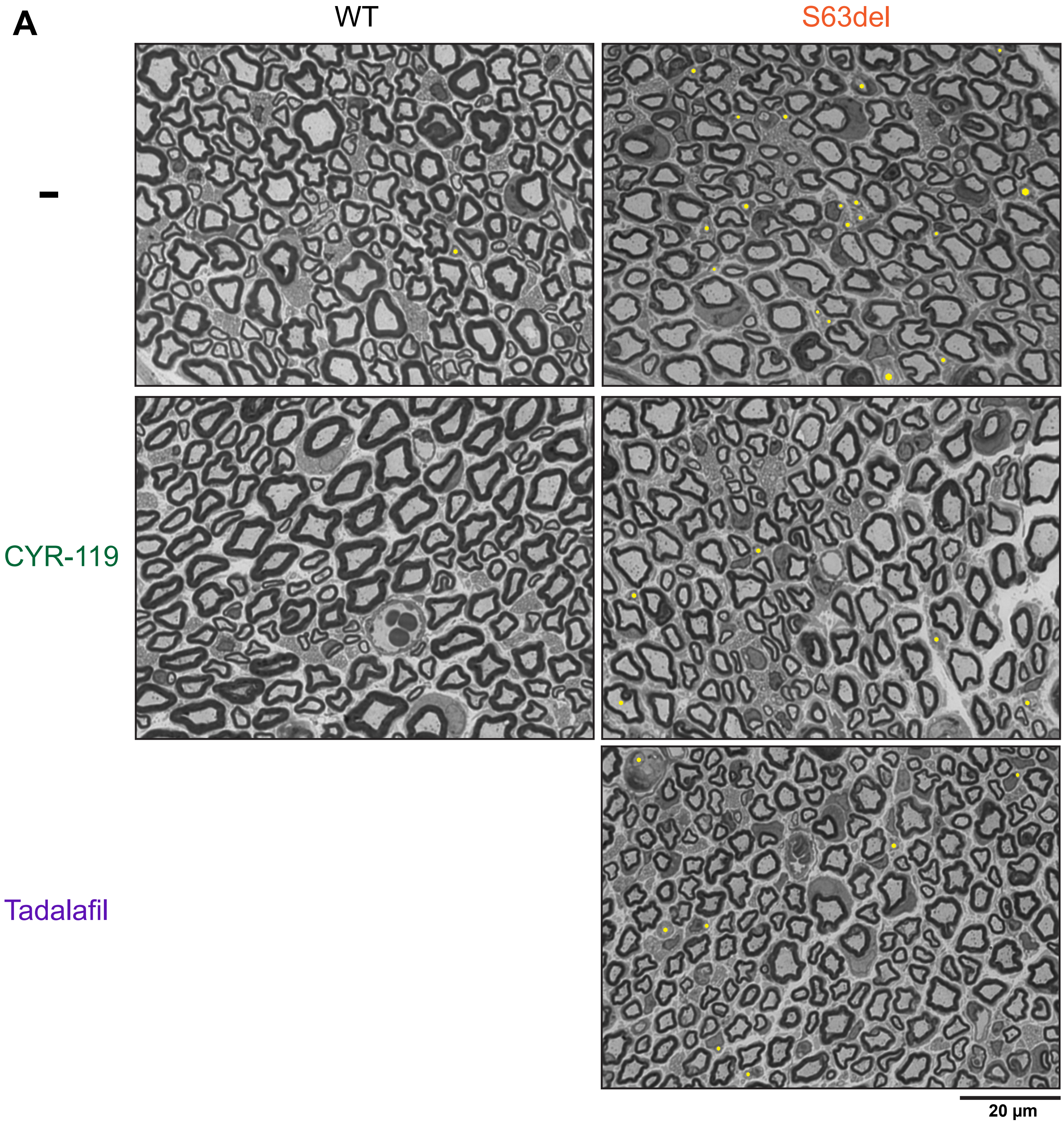

Supplemental Figure 3: Semithin sections from sciatic nerves of WT mice that were treated for 21 days with CYR119 and S63del mice that were treated for 21 days with tadalafil or CYR119

A.) Representative images of semithin sections of sciatic nerves. The 21-day treatment of S63del mice with tadalafil or CYR119 reduced the incidence of unmyelinated fibers, indicated with yellow hexagons.

Supplemental Figure 4

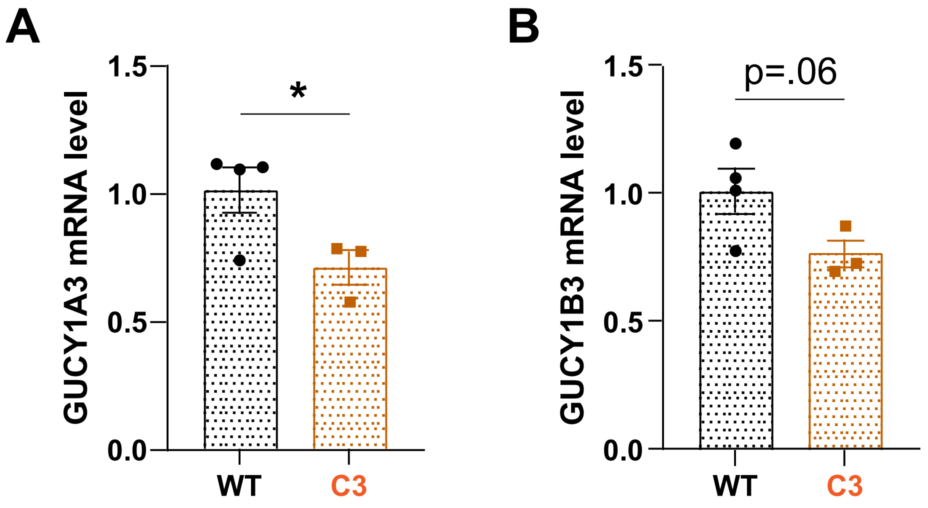

Supplemental Figure 4: Expression of sGC subunits is decreased in the sciatic nerves of C3 mice

A.) mRNA levels of an α subunit of sGC were decreased in the sciatic nerves of C3 mice. Here and below, n=3-4 mice per genotype. Student's t-test. Data were normalized to beta actin and calculated using the $2(-\Delta\Delta Ct)$ method.

B.) mRNA levels of the β subunit of sGC were decreased in the sciatic nerves of C3 mice.

Supplemental Figure 5

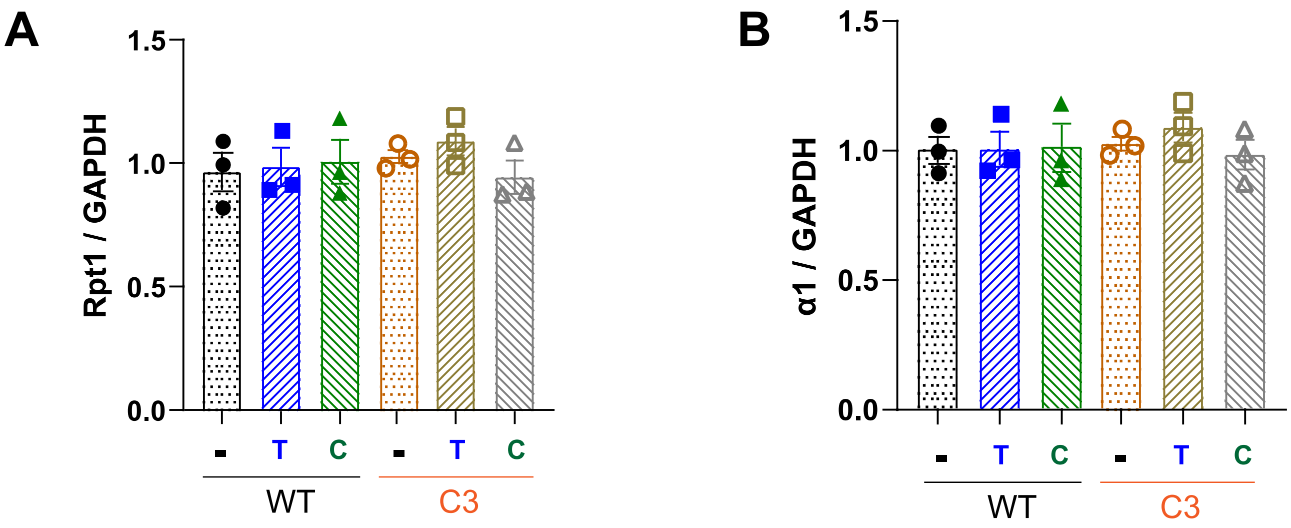

Supplemental Figure 5: Treatment of WT or C3 mice with tadalafil or CYR119 for 7 days does not alter the levels of 26S proteasome subunits in sciatic nerve lysates

A.) Tadalafil or CYR119 treatment for 7 days did not alter the protein levels of the 26S proteasome subunit Rpt1 in WT or C3 mice. Here and below, n=3 mice per genotype, per condition. Error bars represent SEM.

B.) Tadalafil or CYR119 treatment for 7 days did not alter the protein levels of the 26S proteasome subunit α1 in WT or C3 mice.

Supplemental Figure 6

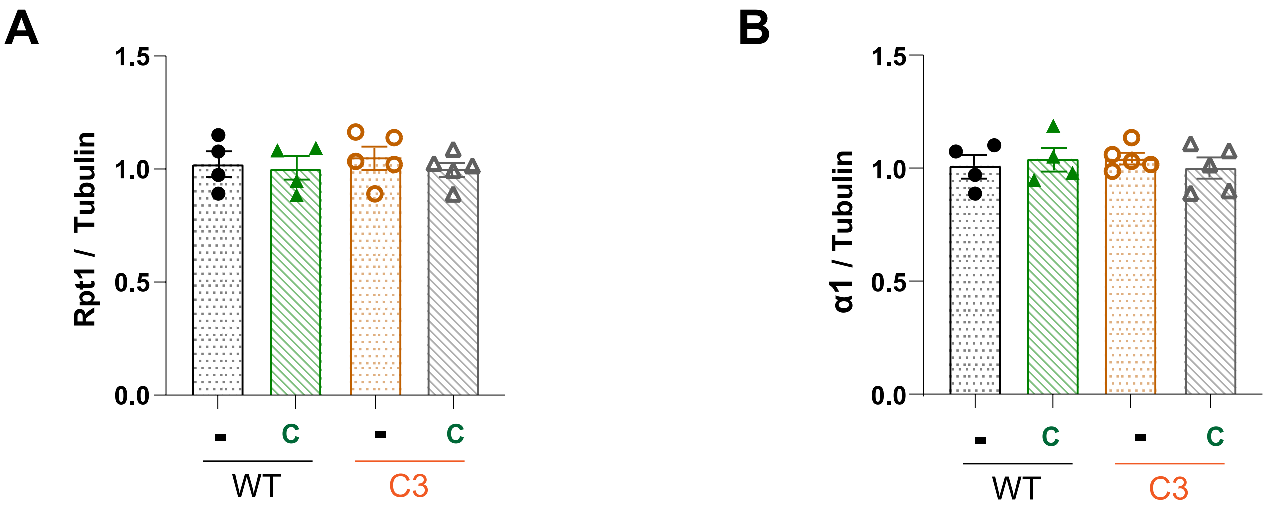

Supplemental Figure 6: Treatment of WT or C3 mice with CYR119 for 21 days does not alter the levels of 26S proteasome subunits in sciatic nerve lysates

A.) CYR119 treatment for 21 days did not alter the protein levels of the 26S proteasome subunit Rpt1 in WT or C3 mice. Here and below, n=4-5 mice per genotype, per condition. Error bars represent SEM.

B.) Tadalafil or CYR119 treatment for 21 days did not alter the protein levels of the 26S proteasome subunit α1 in WT or C3 mice.

Supplemental Figure 7

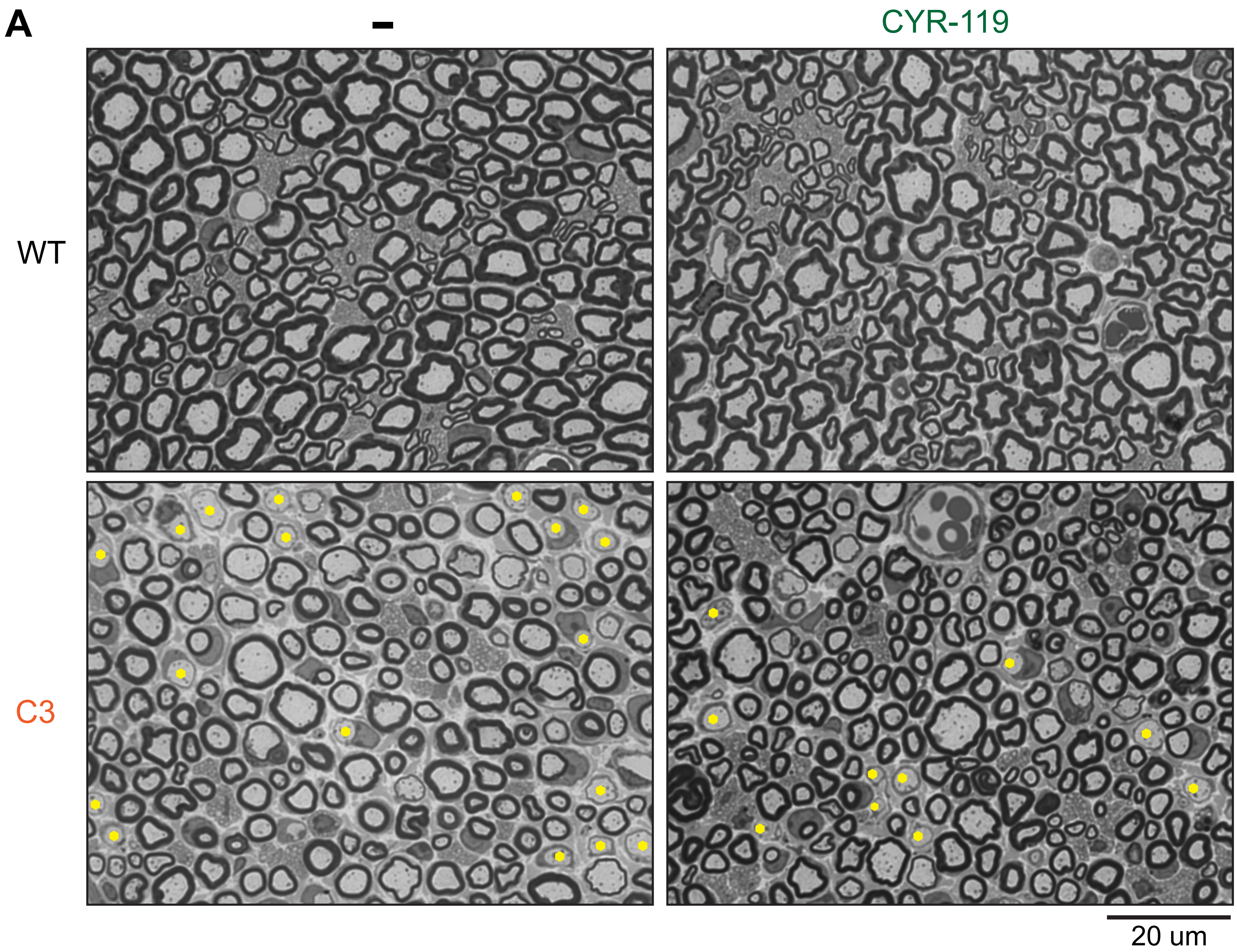

Supplemental Figure 7: Semithin sections from sciatic nerves of WT and C3 mice that were treated for 21 days with CYR119

A.) Representative images of semithin sections of sciatic nerves. The 21-day treatment of C3 mice with CYR119 reduced the incidence of unmyelinated fibers, indicated with yellow hexagons.
